# Effectiveness of treatment for concussion-related convergence insufficiency: The CONCUSS study protocol for a randomized clinical trial

**DOI:** 10.1371/journal.pone.0314027

**Published:** 2024-11-15

**Authors:** Tara L. Alvarez, Mitchell Scheiman, Suril Gohel, Farzin Hajebrahimi, Melissa Noble, Ayushi Sangoi, Chang Yaramothu, Christina L. Master, Arlene Goodman

**Affiliations:** 1 Department of Biomedical Engineering, New Jersey Institute of Technology, Newark, New Jersey, United States of America; 2 Pennsylvania College of Optometry, Salus University, Philadelphia, Pennsylvania, United States of America; 3 Department of Health Informatics, Rutgers University School of Health Professions, Newark, New Jersey, United States of America; 4 School of Applied Engineering and Technology, New Jersey Institute of Technology, Newark, New Jersey, United States of America; 5 Sports Medicine and Performance Center, Children’s Hospital of Philadelphia, Philadelphia, PA, United States of America; 6 Somerset Pediatric Group, Raritan, New Jersey, United States of America; 7 Comprehensive Sports Medicine & Concussion Care, LLC., Bridgewater, New Jersey, United States of America; Aravind Eye Hospital, INDIA

## Abstract

**Purpose:**

To describe CONCUSS, a randomized clinical trial (RCT) designed to compare the following: the effectiveness of immediate office-based vergence/accommodative therapy with movement (OBVAM) to delayed OBVAM as treatments for concussion-related convergence insufficiency (CONC-CI) to understand the impact of time (watchful waiting), the effect of OBVAM dosage (12 versus 16 therapy sessions), and to investigate the underlying neuro-mechanisms of OBVAM on CONC-CI participants.

**Methods:**

CONCUSS is an RCT indexed on https://clinicaltrials.gov/study/NCT05262361 enrolling 100 participants aged 11–25 years with medically diagnosed concussion, persistent post-concussive symptoms 4–24 weeks post-injury, and symptomatic convergence insufficiency. Participants will receive standard concussion care and will be randomized to either immediate OBVAM or delayed (by six weeks) OBVAM. At the Outcome 1 examination (week 7), clinical assessments of success as determined by changes in the near point of convergence (NPC), positive fusional vergence (PFV), and symptoms will be compared between the two treatment groups. After the Outcome 1 visit, those in the delayed group receive 16 visits of OBVAM, while those in the immediate OBVAM group receive four more therapy visits. Outcome 2 assessment will be used to compare both groups after participants receive 16 sessions of OBVAM. The primary measure is the between-group differences of the composite change in the NPC and PFV at the Outcome 1 visit. Secondary outcome measures include individual clinical measures, objective eye-tracking parameters, and functional brain imaging.

**Conclusions:**

Major features of the study design include formal definitions of conditions and outcomes, standardized diagnostic and treatment protocols, a delayed treatment arm, masked outcome examinations, and the incorporation of objective eye movement recording and brain imaging as outcome measures. CONCUSS will establish best practices in the clinical care of CONC-CI. The objective eye movement and brain imaging, correlated with the clinical signs and symptoms, will determine the neuro-mechanisms of OBVAM on CONC-CI.

## Introduction

Concussion is considered a substantial health problem among adolescents and young adults in the United States [1–4]. While some symptoms resolve with supportive care, results of a longitudinal study showed that 41% of the 591 concussed patients were classified as having persistent post-concussive symptoms (PPCS), where symptoms do not dissipate after two weeks [5]. Another study reported that 46% of the 2946 enrolled children were diagnosed with PPCS two weeks post-injury, which decreased to 33% four weeks post-injury [6]. PPCS profoundly impacts a person’s ability to function scholastically [7–11] and professionally [12–15]. Adolescents are particularly vulnerable to cognitive and developmental concussion consequences, often having a prolonged recovery and poorer outcomes than adults [11,16]. Among various conditions associated with PPCS, oculomotor dysfunction is prevalent and considered one of the phenotypes of concussion [17–21]. Symptoms of oculomotor dysfunction include dizziness, headaches, eye strain, discomfort while reading, inability to sustain attention during a long-duration visual task, experiencing fatigue faster post-injury compared to pre-injury, difficulty with attention and memory, and blurry/double vision [4,22–24].

Convergence insufficiency (CI) is associated with many of the aforementioned characteristic symptoms of PPCS [23,25–28]. In one study of PPCS, 70.4% of 260 concussed athletes reported visual disturbances, the seventh most frequently reported symptom [29]. CI is the most common non-strabismic binocular vision disorder in children and young adults, with prevalence estimates from school-based populations ranging from 3.4% to 12.7% for typically occurring CI (TYP-CI) [30–35]. However, TYP-CI is not associated with concussion. Much higher rates of CI have been reported in children and adults [27,36] with PPCS, with estimates of 38% to 49% for children [28,37] and 23% out of 500 adults [38]. Since concussion-related CI (CONC-CI) is associated with a traumatic acceleration of the head resulting in injury, this suggests a different etiology than TYP-CI, which presumably develops over time and is not associated with head injury.

Clinicians use the same diagnostic criteria for TYP-CI and CONC-CI, such as receded near point of convergence, inadequate positive fusional vergence, and a greater exophoria at near compared to far. The greater prevalence of convergence insufficiency, frequent comorbidity of vestibular dysfunction, and acute post-injury onset [27] suggest that CONC-CI may be a unique condition compared to TYP-CI. To better understand the underlying neuro-mechanisms of CONC-CI, CONCUSS integrates objective eye movement recording, advanced brain imaging methodologies, such as functional Magnetic Resonance Imaging (fMRI), and clinical signs and symptoms to develop a holistic understanding of how CONC-CI differs from binocularly normal controls and TYP-CI. More importantly, the longitudinal CONCUSS assessments will discover the neuro-mechanistic changes evoked by office-based vergence accommodative with movement (OBVAM) therapy in those with CONC-CI.

Diagnostic protocols and therapeutic interventions are well-established for TYP-CI [39–43]. Previous studies integrating task-induced and resting-state fMRI have answered important questions regarding the brain regions that show reduced functional activity and altered resting-state functional connectivity for TYP-CI compared to binocularly normal controls [44,45]. Results from multiple clinical trials summarized in a Cochrane network meta-analysis [46,47] show that office-based vergence accommodative therapy (OBVAT) is an effective treatment in TYP-CI children [40,48,49] and young adults [39,50,51]. Prior results discovered the neuro-mechanistic effects of OBVAT, leading to more effective rehabilitation strategies in TYP-CI [44,52–55]. OBVAT was modified to OBVAM because of the high co-morbidity of vestibular dysfunction in CONC-CI and common symptomology of dizziness and nausea [56,57]. Previous research showed that office-based vergence and accommodative therapy are the most effective treatments for remediating symptoms and improving visual function in pediatric [40,48,49] and adult [50,51] individuals with CI.

No scientifically validated treatment for CONC-CI has been assessed [58]. The differences in prevalence, mode of onset, such as longstanding versus acute, and severity of the condition have led to a debate about whether the diagnostic and management procedures effective for TYP-CI should be utilized for CONC-CI. Independent groups have published pilot studies investigating only a single treatment arm in CONC-CI and suggest that the same 12 one-hour sessions of OBVAT that are effective for TYP-CI may improve the visual function of those with CONC-CI [59–61]. An adequately powered randomized clinical trial (RCT) investigating the effectiveness of vergence/accommodative therapy for CONC-CI, comparing different doses of treatment, and providing an understanding of the neuro-mechanism by which therapeutic interventions may remediate symptoms and improve vergence function is not available. The CONCUSS RCT addresses these unanswered questions and will provide evidence to support best practices in CONC-CI care.

To address these gaps, the CONCUSS RCT’s primary aim is to answer whether vergence/accommodative therapy is an effective treatment for CONC-CI by comparing the immediate OBVAM group to the group in the six weeks of watchful waiting who have not yet begun OBVAM. The other aims include the following: 1) if OBVAM is effective then which is a more effective dose -12 or 16 one-hour vergence/accommodative therapy sessions to restore normal oculomotor function and remediate symptoms for those with CONC-CI. 2) Does a further delay of six weeks impact the effectiveness of vergence/accommodative therapy compared to immediate interventions? 3) What underlying neuro-mechanism(s) would explain the effectiveness of vergence/accommodative therapy for the CONC-CI population? CONCUSS will test the hypothesis that the changes in clinical measures, symptoms, objective oculomotor function, and imaging results will be significantly better in participants in the immediate vergence/accommodative therapy treatment group than in the delayed treatment group at Outcome 1. CONCUSS will also test the hypothesis that vergence/accommodative therapy administered to CONC-CI will improve neuroimaging stimulus-induced and resting-state measures that correlate to the improved maximum speed of convergence eye movement, convergence endurance, clinical signs, and symptoms.

## Methods

### Study design

A multi-site longitudinal, double-armed, parallel randomized controlled design will be conducted per the CONSORT 2010 agreement [62]. Standardized diagnostic protocols used in the previous studies, including CITT [43], CITT-ART [40], and CINAPS [39], will be utilized in the CONCUSS RCT.

**Fig 1:**
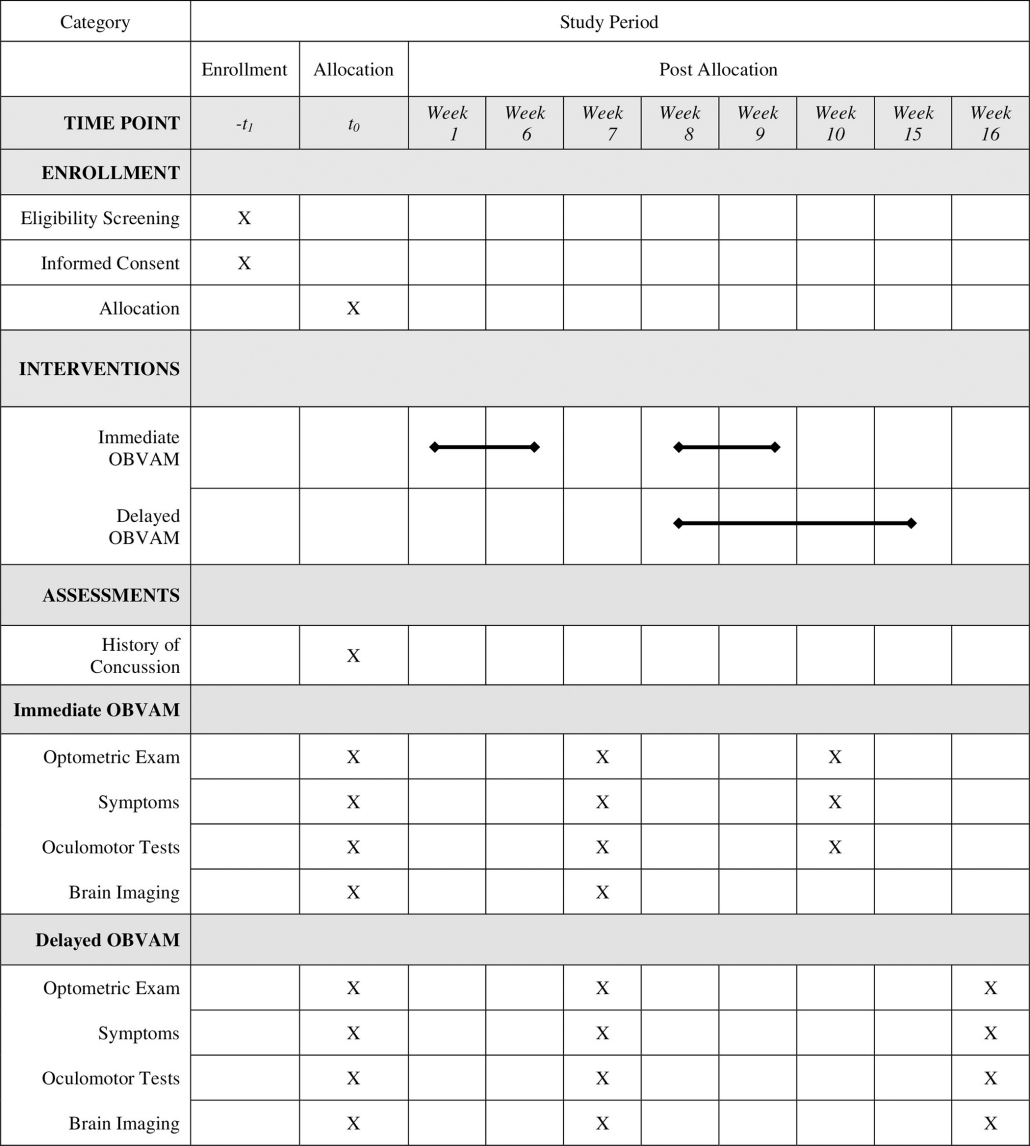
The SPIRIT schedule of enrollment, interventions, and assessments.

**Fig 2:**
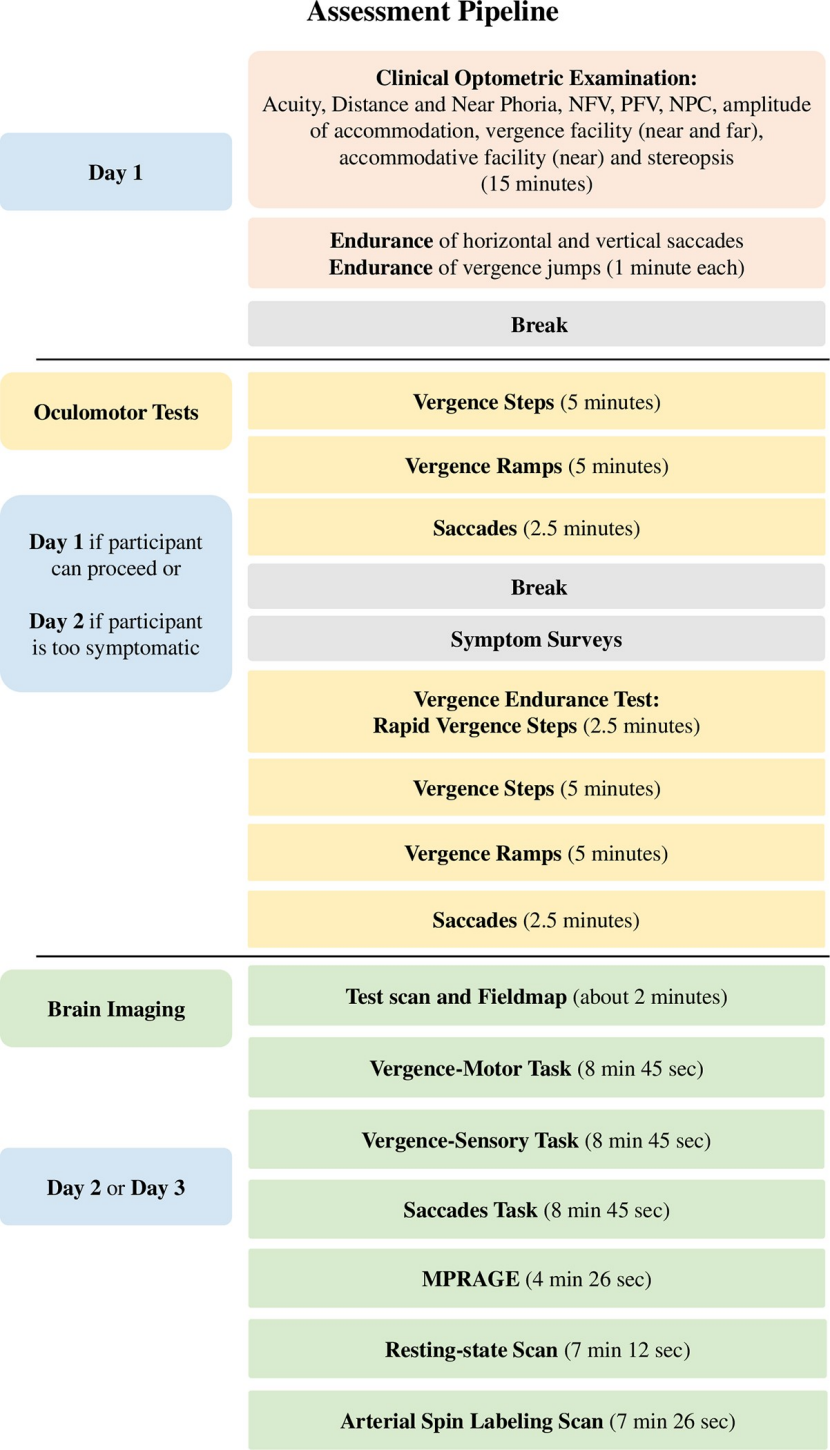
Assessment pipeline showing the order of optometric tests, the amount of time per task, and the day the task is completed.

The CONC-CI population is highly symptomatic, so fewer experimental tasks will be conducted to minimize participants’ discomfort than procedures implemented in the past CINAPS study [39,44]. Physicians specializing in concussion in adolescents will identify participants with a medical diagnosis of concussion. Concussion diagnoses follow the Consensus Statement on Concussion in Sports [63]. Potential participants will be screened for eligibility before enrollment in the study. Eligibility consent will be obtained before the optometrist performs the examination administered by research coordinators. Adults will sign a written consent. Children less than 18 years of age will sign written assent, and their legal guardian will sign a written consent. If the participant is a child, then both consent and assent are required for enrollment. All participants will be asked for approval to allow future analysis of de-identified data posted to public repositories. The optometric clinical exam will take about 15 to 30 minutes, depending on the symptom level of the participant. If the participant is not eligible for CONCUSS, they will be referred to their physician, and the optometric exam results will be transmitted to their concussion doctor. If the participant is eligible, written consent/assent will be obtained. If the participant enrolls, they will be randomly allocated to immediate OBVAM or delayed treatment. It is anticipated that most participants will be symptomatic and return for objective eye movement recording during a separate session. If the participant can proceed, then eye movement recording can be done on the same day as the optometric exam. Functional brain imaging will be on a different day. The same testing performed at enrollment will be re-administered as the Outcome 1 visit (clinical oculomotor examination, symptoms surveys, eye movement recordings, and functional imaging). Outcome 2 includes all participants’ clinical oculomotor examination and objective eye movements. Functional imaging for Outcome 2 will only occur in the delayed vergence/accommodative therapy group after the completion of 16 sessions of therapy. After the enrollment assessments (two sessions of about one hour each), 100 CONC-CI participants will be randomly assigned to immediate or delayed therapy. The SPIRIT schedule of enrollment, interventions, and assessments is presented in Fig 1. A schematic pipeline of all measurements in each session is presented in Fig 2.

Both treatment groups will receive standard concussion care (SCC) administered by their physician.

**Fig 3:**
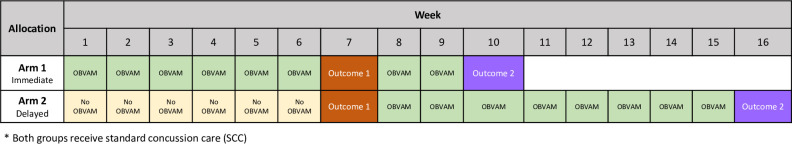
RCT timeline showing when assessments are performed and when OBVAM sessions are conducted per week.

Given the high prevalence of dizziness and nausea in CONC-CI [27,28], OBVAT will be modified from previous studies of children and adults with TYP-CI [39,40,43] to include head and body movement in some procedures. From here forward, the treatment will be referred to as OBVAM. Fig 3 illustrates the flow of enrollment, assessment outcomes, and therapy for the two treatment groups.

In the immediate OBVAM group, participants will receive two therapy sessions per week for six weeks (12 sessions) along with SCC. Participants in delayed OBVAM will only receive SCC for the first six weeks. The Outcome 1 visit will be scheduled for both groups in the 7th week. Afterward, participants in the immediate OBVAM group will receive an additional four sessions of OBVAM (totaling 16 sessions) and will be reassessed after two weeks (week 10). After the Outcome 1 examination at seven weeks, participants in the delayed OBVAM group will receive two sessions of therapy per week for eight weeks (16 sessions) with SCC and will be reassessed after OBVAM. Weekly reminders for therapy sessions or assessments will be given by the research coordinators to improve participant retention and reduce deviations from the study protocol. Participants in both groups will be assessed in a 1-year follow-up for all the measurements. The protocol of this study is registered in ClinicalTrials.gov (Identifier: NCT05262361; March 2, 2022, https://clinicaltrials.gov/study/NCT05262361). The study start date is September 1, 2021. The recruitment for both the NJIT and CHOP clinical sites is February 21, 2022. The trial is designed in coherence with the Standard Protocol Items: Recommendations for Interventional Trials (SPIRIT) [64].

### Participants

Participants will be recruited from two medical settings and screened for eligibility based on the inclusion and exclusion criteria listed in Table 1. One medical setting is a pediatric private practice of a physician specializing in pediatric concussion (AG). The second site is the Minds Matter Concussion Program at the Children’s Hospital of Philadelphia (CLM). Participant recruitment is planned for about four years. Participants will all receive therapeutic OBVAM intervention at no cost. All participants will be between the ages of 11 and 25 years old with a medical diagnosis of concussion 4 to 24 weeks post-injury with persistent post-concussive symptoms and will be screened for eligibility. A history of multiple concussions will be permitted, and a medical history of when prior concussion(s) occurred, mechanism of injury, and symptomology post each concussion will be recorded. Inclusion and exclusion criteria for CONC-CI participants are presented in Table 1.

**Table 1:** Study eligibility and exclusion criteria.

Eligibility Criteria
1. Medical diagnosis of concussion and 4 to 24 weeks post-injury 2. Age 11 to 25 years 3. CI Symptom Survey (CISS) score ≥ 16 for children 11–17 years, ≥ 21 for adults 18–25 years 4. Receded near point of convergence of ≥ 6 cm as the break point 5. Insufficient positive fusional vergence at near (i.e., failing Sheard’s criterion or positive fusional vergence ≤15Δ base-out break) 6. Best-corrected distance and near visual acuity of 20/25 or better in each eye 7. Local stereopsis of 70 seconds of arc or better and random dot stereopsis of 500 seconds of arc or better 8. Wearing appropriate refractive correction (spectacles or contact lenses) for at least two weeks before final determination of eligibility for any spherical equivalent refractive error of ≥2.00 D of hyperopia, ≥1.00 D of myopia, ≥1.00 D of anisometropia, or ≥1.5 D of astigmatism based on subjective refraction 9. Refractive error corrections adhered to the following guidelines: full hyperopic sphere power or symmetrically reduced by no more than 1.50 D, spherical equivalent myopia and spherical equivalent anisometropia within 0.75 D of full correction; and astigmatism within 0.75 D of full correction and axis within 6 degrees for magnitudes of ≥ 1.00 D. 10. Not wearing base-in prism or plus add at near for two weeks before study enrollment and the duration of the study 11. Understanding of the protocol and willingness to accept randomization
**Exclusion Criteria**
1. Constant strabismus at distance or near 2. Vertical heterophoria ≥ 2Δ at distance or near 3. ≥ 2-line interocular difference in best-corrected distance visual acuity 4. Manifest or latent nystagmus 5. History of prior strabismus, intraocular, or refractive surgery 6. CI previously treated with any form of office-based vergence/accommodative therapy or home-based vergence therapy (e.g., computerized vergence therapy)
7. Diseases known to affect accommodation, vergence, or ocular motility, such as multiple sclerosis, Graves orbitopathy, myasthenia gravis, Parkinson’s disease 8. Pregnancy or planning on becoming pregnant during the study time frame

### Procedures and data collection

A team of study-certified clinicians and researchers will be responsible for recruitment and data collection at the study enrollment baseline and outcome examinations. While many concussion studies refer to baseline as the preinjury measurement, we choose to use the word baseline as the study enrollment timepoint to be consistent with other RCTs. Study data will be collected and managed using REDCap electronic data capture tools hosted at New Jersey Institute of Technology (NJIT) and Children’s Hospital of Philadelphia (CHOP) [65,66]. REDCap (Research Electronic Data Capture) is a secure, web-based software platform designed to support data capture for research studies, providing 1) an interface for validated data capture; 2) audit trails for tracking data manipulation and export procedures; 3) automated export procedures for data downloads to common statistical packages; and 4) procedures for data integration and interoperability with external sources. REDCap will be architected before any participant enrollment with a blocked randomization sequence developed by a statistician. The randomization was programmed into REDCAP to ensure an approximately equal number of participants in each arm. The clinicians performing assessments cannot determine which participants will be assigned to each arm. Participants will be assigned to groups 1 or 2 with no more than four sequential allocations to one arm using a random seed generator in MATLAB. The optometrist conducting the clinical optometric exams will be masked from the therapy type. The researchers who analyze the quantitative eye movements and imaging data will also be masked to the treatment group during preprocessing and individual analyses. Masking will diminish conscious and subconscious biases, which can occur while studying a targeted population [67,68]. Participants, research coordinators, and therapists will not be masked to the intervention type that the participants receive due to the nature of the study. Data analysts will be unmasked to conduct group-level analyses. Once data collection begins, monthly audits will be conducted to minimize any missing data.

### Primary outcome measure

The primary outcome measure is the between-group differences and a 2-sided 95% confidence interval of the composite change in the near point of convergence (NPC) and positive fusional vergence (PFV) at the Outcome 1 visit in week 7 (after six weeks of treatment in the OBVAM group and six weeks of SCC only in the delayed OBVAM group) and will be calculated using an intent-to-treat analysis. Based on the changes in both the NPC and PFV data, the participant outcome will be categorized as "successful," "improved," or "non-responder." A successful outcome will be a participant who meets the following criteria: 1) normal NPC (<6cm) *and* an improvement of ≥4 cm and 2) normal PFV (met Sheard’s criterion and break value >15Δ) *and* an improvement of ≥10Δ. A participant will be classified as having an improved outcome if the NPC *and* PFV are either normal *or* improved. A participant will be classified as a non-responder when at least one of the two clinical measures (NPC or PFV) is neither normal *nor* improved.

### Secondary outcome measures

For the secondary measures, a comparison will be made for the following clinical signs: changes in NPC, PFV, and the CISS score between the two groups from enrollment in the Outcome 1 visit. A within-group analysis will determine if there was any benefit to the four additional therapy sessions administered to the OBVAM group participants after the Outcome 1 visit. Secondary measures will also include a change in symptoms by comparing the CISS between "pre-injury" and Baseline (study enrollment), Outcome 1 and Outcome 2. The change in the CISS score from post-injury Baseline to Outcome 1 and post-injury Baseline to Outcome 2 will be evaluated.

Secondary Outcome measures also include the objective eye movement recording and brain imaging data. Objective eye movement outcome measurements include vergence peak velocity [69], the endurance of vergence eye movements, or the ability to maintain vergence peak velocity over a sustained vergence task [70]. The following functional imaging results will be assessed as secondary outcome measurements: functional activity within the vergence neural circuit for peak activation, the spatial extent of vergence neural substrates, and whole-brain functional connectivity.

### Assessment methods

#### Clinical optometric exam measures

Best-corrected visual acuity will be assessed for each eye at a distance using the Snellen eye chart. Subjective refraction will be performed to ensure that participants meet the refractive error inclusion criteria. The cover-uncover (unilateral cover) test and prism and alternate cover test (PACT) will be performed at distance (4 m) and near (40 cm) to objectively determine the direction and magnitude of the deviation. Participants will wear their refractive correction (if prescribed) and be asked to keep a 20/30 isolated letter clear during testing. For PACT testing, a prism will be added until the direction of movement is reversed (e.g., exophoric becomes esophoric). The magnitude will be recorded as the largest prism that results in neutrality (before the first reversal movements, i.e., high neutral). The Randot Stereo test (Stereo Optical, Chicago, IL, USA) will assess local and global stereopsis. A local stereopsis of 70 seconds of arc or better and a global stereopsis of 500 seconds of arc or better are required to enroll. Assessments are summarized in Fig 2.

Other oculomotor testing will include the following conventional optometric exam parameters. The Astron Accommodative Rule (Bernell, Mishawaka, IN, USA item GR50, Bernell) will be used to measure the NPC. The device consists of a rod with a movable slide, and a single column of letters (20/30 equivalent at 40 cm) will be used as a target. PFV and negative fusional vergence (NFV) will be measured using the step vergence procedure with a handheld horizontal prism bar (Gulden Ophthalmics Elkins Park, PA, USA, Product Number 11112). Negative fusional vergence will be measured before positive fusional vergence. The participant will view a 20/30 size column of letters (Gulden Ophthalmics Elkins Park, PA, USA, Product Number: 15302) held on the participant’s midline at 40 cm. The examiner will move the prism bar to achieve a change at a rate of ~2 Δ /sec. The participant will report when the target is blurry or double and when they can regain fusion. The vergence facility will be assessed with a 12BO / 3BIΔ prism (Gulden Ophthalmics Elkins Park, PA, USA, Product Number: 11107) and the Gulden Fixi-Tic (Gulden Ophthalmics Elkins Park, PA, USA, Product Number: 15112) at near (40 cm) and repeated at far (4 m). The 12BO / 3BIΔ prism will be placed before the right eye (base-out first), and when the participant experiences single, clear vision, they will say "now." The base-in prism is then placed before the right eye, and once the participant experiences single and clear vision, they again say "now." The procedure is repeated for one minute. The examiner will record the number of times a participant can see the target as single and clear in one minute.

Accommodation will be assessed using the following methods. The monocular amplitude of accommodation will be evaluated for both the right and left eye with the Astron Accommodative Rule using a vertical column of 20/30 letters placed initially at 40 cm and slowly moving towards the participant at a rate of 1cm/sec until it blurs. The participant will be instructed to keep the letters clear and tell the examiner when the letters first blur (first sustained blur). The monocular accommodative facility will be assessed using a ±2D lens flipper (Gulden Ophthalmics Elkins Park, PA, USA, Product Number: 11132) and the Gulden Fixi-Tic target for the right eye only. The participant will view the Gulden fixation target at 40 cm. The examiner will alternate between each lens when the participant states the visual target is clear. The number of lens flips will be counted within one minute.

#### Visual and concussion symptoms

Symptoms surveys include the Convergence Insufficiency Symptoms Survey (CISS) and VisQual-T [71]. CISS is a 15-question survey to assess visual comfort while reading. The greater the score, the more symptomatic the participant. A participant is classified as symptomatic using a threshold score of ≥21 for adults or ≥16 for children. The CISS has a sensitivity of 97.8%, a specificity of 87%, and an interclass correlation coefficient of 0.89 [72,73]. The Vision Quality of Life with Time (VisQuaL-T) is a series of 10 questions about daily activities inquiring about the duration of time when visual symptoms become apparent. VisQuaL-T has an ICC = 0.83 within normative data [71]. All participants will be asked to complete the CISS and VisQual-T surveys two times at enrollment, once recalling their symptoms pre-injury, and a second time based on how they felt in the past few days. At Outcome 1, Outcome 2, and the one-year follow-up assessment, these surveys will only be administered once.

#### OculoMotor Assessment Tool (OMAT)

The OculoMotor Assessment Tool (OMAT) (Gulden Ophthalmics Elkins Park, PA, USA, Product Number: 18009 and the OMAT smartphone application) will be used to assess horizontal saccades, vertical saccades and vergence jumps [74]. Each of these tests lasts one minute. The OMAT app, available for a smartphone, enables the user to count and time the number of eye movements for the initial and later 30 seconds of the one-minute test. The OMAT provides data that can be used to assess oculomotor endurance. Before using the OMAT, participants will be asked to rate the following symptoms: headache, dizziness, nausea, and fogginess on a Likert Scale from 0 to 10 (zero is no symptoms, and ten is highly symptomatic). The symptoms will be rated again after each of the OMAT subtests.

The OMAT will be placed on the participant’s nasion for horizontal and vertical saccades. The participant will be asked to look from one “X” placed 25 cm along the midline and move their eyes from 25⁰ in the left visual field to 25⁰ in the right visual field (50⁰ horizontal saccade). The participant will be asked to make as many saccades as possible in one minute and to slow down or even stop if symptoms become too overwhelming. For vertical saccades, the OMAT will then be rotated for the participant to initiate 50⁰ vertical saccades. For vergence jumps, two targets (vertical lines) will be added to the OMAT bar placed along the midline at 9 cm and another at 24 cm to stimulate a symmetrical vergence step stimulus of 20° (35 Δ). For each task, the number of eye movements will be counted using the OMAT application and recorded in REDCap.

#### Objective eye movement recordings

An Oculus DK2 virtual reality headset integrated with ISCAN (Woburn, MA, U.S.A.) infrared (λ = 950nm) video-based eye tracker with a sampling rate of 240 Hz and a manufacturer-reported resolution of 0.1⁰ will be utilized. Hygiene masks will be used so the virtual reality headset does not touch any participants. The visual stimulus will be a Distribution of Gaussian with a 2⁰ eccentricity for mostly foveal stimulation.

The objective eye movement recordings will comprise two tests: an oculomotor battery and an oculomotor endurance test with a break between tests. The battery will contain eight monocular calibrations (four for each eye) located at known angles of 1⁰, 3⁰, 5⁰, and 7⁰ inward rotation, encompassing the visual field of the eye movements to be recorded. Then, convergence and divergence symmetrical steps of 4⁰ and 6⁰ magnitude changes after a delay of up to 0.5 sec to reduce predictive cues [75] known to stimulate the dorsal lateral prefrontal cortex [76] will be presented over a 5-minute duration. There will be 30 convergent steps starting at an initial vergence demand of 4⁰ and ending at a vergence demand of 8⁰ and 30 divergent steps beginning at an initial vergence demand of 8⁰ and ending at a vergence demand of 4⁰. There will be ten convergence and divergence 4⁰ steps between the vergence demands of 6⁰ and 10⁰. There will be ten convergence and divergence 6⁰ steps between the vergence demands of 4⁰ and 10⁰. All vergence steps will be recorded for a 5-second duration. Then, slow (1⁰/sec) and fast (4⁰/sec) convergence and divergence ramps will be presented randomly for a 5-minute total duration. There will be 15 observations of each ramp type between the vergence demand range of 4⁰ to 10⁰. The last 2.5 minutes of the test will be 5⁰ and 10⁰ leftward and rightward saccades presented pseudo-randomly over the range of 5⁰ into the left visual field from midline to 5⁰ into the right visual field from midline. Saccades will be presented for 2 seconds for each observation, where there will be 30 observations of saccades of 5⁰ magnitude and 20 observations of saccades with a 10⁰ magnitude. Participants will be given breaks between vergence steps, vergence ramps, and saccades. The duration of each break will be participant-dependent due to potential concussion-related symptoms. There will be a total of 12.5 minutes of eye movement recording for the oculomotor battery test.

Oculomotor endurance, or the ability to sustain performance, has been suggested to be an independent risk factor post-concussion [70]. Hence, the second test is an oculomotor endurance test. It will have the same eight monocular calibrations of four per eye at the same positions as the oculomotor battery test. Then, there will be 2.5 minutes of only 4⁰ convergence and 4⁰ divergence symmetrical steps between a vergence demand of 4⁰ to 8⁰ for a 3-second duration. There will be 25 observations of convergence and 25 divergence steps for a total of 50 vergence movements. Then, the oculomotor battery described above will be repeated: 5 minutes of vergence steps, 5 minutes of vergence ramps, and 2.5 minutes of saccades. The vergence endurance test (VET) is 15 minutes of eye movement testing. The break between each movement type will be participant-dependent based on how symptomatic they may feel. It is also anticipated that not all participants will be able to complete testing due to symptoms.

#### Functional Magnetic Resonance Imaging (fMRI)

A 3 Tesla Siemens Prisma with a 64-channel head coil (Siemens Medical Solutions, Parkway Malvern, PA, U.S.A.) will be used at the Rutgers University Brain Imaging Center (RUBIC) for the participants within the NJ site. An Eyelink-1000 system (SR Research, Canada) will record the right-eye movements at 250 Hz to ensure that the participant performs the requested oculomotor tasks and is calibrated before image acquisition using a 9-point calibration. A mirror on the head coil, angled in front of the eyes and situated 15 cm away from the participant’s nasion, will allow the participant to view a 1920 by 1080-pixel resolution screen 80 cm from the mirror. Participants needing eyeglasses will use fMRI-compatible glasses to the closest 0.5 D of their refractive prescription throughout the session, calculated as the mean sphere plus half of the cylinder, or wear their contacts which is preferred for refractive correction. The details of the tasks and what will occur while being scanned will be explained to each participant before starting the experiment. All females who have the possibility of being pregnant will take a pregnancy test or sign a waiver confirming that they are not pregnant. No pregnant females will be scanned. A detailed screening form will be administered to all participants before every scan to ensure that no ferrous metal, implants, or conditions that can lead to an adverse reaction will occur while being imaged. An emergency squeeze ball is also available to alert the MRI technician if the participant is distressed and needs to stop the experiment.

After the localizer scan of 14 seconds and gradient field map recording of 1 minute 18 seconds, three task-induced fMRI recordings will be conducted. First, a 15-second echo planar imaging (EPI) scan will be done so participants are less startled by the sounds of the fMRI machine. The tasks include a vergence-motor task (VM), a vergence-sensory task (VS), and a saccadic task. All stimulus tasks will be 25 seconds per block, with eleven rest blocks and ten blocks of task. The stimulus-induced tasks will be 8 minutes and 45 seconds in length each. A magnetization-prepared rapid acquisition gradient echo (MP-RAGE) will be next, which will take 4 minutes and 26 seconds. Then, a resting state scan (rs-fMRI) will be conducted for 7 minutes 12 seconds, and arterial spin labeling (ASL) scans for 7 minutes 26 seconds. The total imaging time is 54 minutes, and if participants need more of a break between tasks, the ASL scan will be dropped.

Due to the symptomatic behavior of the CONC-CI participants, the three stimulus-induced tasks (VM, VS, and saccade) that need more visual attention from the participants are planned for the beginning of the fMRI scanning queue to reduce any probable effects of fatigue on the expected brain activations and blood-oxygen-level-dependent (BOLD) signals. Functional imaging experiments for the three stimulus-induced and the resting state will be acquired using an echo planar imaging (EPI) sequence with the following parameters: 720 ms for TR, 33 ms for TE, 90° flip angle, 192 mm FOV, 3.0x3.0x3.0 mm^3^ spatial resolution, and 56 slices in an axial configuration. The disparity vergence step experiment evokes the afferent and efferent portions of the vergence motor (VM) system. It will use a set of eccentric squares that stimulate 4° and 6° symmetrical disparity vergence step responses analogous to the quantitative eye movement experiment. The procedure is analogous to a clinician asking a patient to look between two targets along the participant’s midline. The stimulus target extends 2° by 2° in stimulus eccentricity. The vergence step stimuli will be presented for a duration between 1.8 to 2.8 seconds per eye movement using the following order: 3° Convergent (Con), 3° Con, 3° Divergent (Div), 4° Con, 2° Div, 3° Con, 2° Div, 3° Div, and 3° Div steps. The vergence sensory (VS) experiment will be the same as the VM experiment, with a dot in the center of the screen added, but the participants will be asked not to move their eyes while the eccentric squares move on the screen. This task would exclude the motor response associated with the vergence motor and stimulate the BOLD signals related to the sensory vergence system. The VM protocol has a good intraclass correlation (ICC>0.4) coefficient for stimulating the vergence neural substrates [77]. The protocol will be repeated for saccades where the rest block is a small square target that is stationary on the participant’s midline for 25 seconds, followed by 5° or 10° saccades into the left or right visual field and be a square target similar to prior studies [78,79].

The MPRAGE sequence will be used to acquire a high-resolution anatomical reference with the following parameters: 1900 ms for time repetition (TR), 2.52 ms for time echo (TE), 900 ms for longitudinal relaxation time (T1), 9° flip angle, 256 mm field of view (FOV), 1.0x1.0x1.0 mm^3^ spatial resolution, and 176 slices within an axial orientation. During the rs-fMRI scan, participants will be asked to keep their eyes open and not think about or concentrate on any particular thoughts using the same EPI sequence parameters used during the stimulus-induced tasks. The last scan will be the ASL, which will have the following parameters: a TR of 4600 ms, TE of 16.18 ms, 46 slices, a FOV of 192 mm, a flip angle of 180° a slice thickness of 3 mm. Each participant will be asked to close their eyes in the ASL scanning.

### Treatment methods

OBVAT is well-documented in many prior studies [39,40,43] and has four phases. OBVAM is OBVAT with the addition of movement procedures. The first phase will be designed to manage some of the visual/movement symptoms common in CONC-CI using eye movements associated with head and body movement. In phase 2, techniques will stimulate gross convergence, and positive fusional vergence using Brock string, Vectograms, and Computer Orthoptics. Monocular accommodative therapy will also be emphasized in Phase 2 using Accommodative Lens Rock. Once participants can perform these tasks and reach the endpoint criteria, they will move to phase three, which includes ramp fusional vergence and monocular accommodative therapy with Vectograms, Aperture Rule, and Accommodative Lens Rock. The final phase comprises jump fusional vergence and binocular accommodative facility. CONCUSS will replicate the program used in the CITT with the addition of incorporating movement into the eye movement and vergence procedures, including lateral and vertical movement of the head, standing, walking forward and backward, walking in a circular pattern, and rotating in a circle while engaged in vergence jump and ramps. The complete manual of procedure details of the OBVAM protocol can be found at https://centers.njit.edu/vision/OBVAM/.

A home computer vision therapy program (HTS https://htsvision.com/hts2/) will be utilized for the home reinforcement. HTS stimulates disparity convergence and sequences the therapy from ramp to step vergence demands, always providing immediate and accurate feedback to the participant [80]. The HTS software also allows the investigator to monitor compliance and performance with the prescribed treatment by logging the number and time of sessions. Brock string and eccentric circles exercises will also be performed. Participants will be instructed to work with these home exercises thrice per week for about 10 minutes each time OBVAM is not performed in the office.

Standard-Community Concussion Care (SCC) will consist of a return to learn/school and a return to play (if applicable) plan. Physical exercise will be initiated once the initial symptoms have begun to improve. Patients involved in sports will be cleared for contact or collision sports once their symptoms return to pre-injury values; they are asymptomatic at school without accommodations and can do heavy physical exertion without symptoms. Based on the clinical exam results, patients may be given neck stretches, saccades, vestibular ocular reflex (VOR), or balance exercises. Participants may be given smooth pursuit exercises based on the clinical exam measurement. For participants with vestibular signs and symptoms or cervical neck dysfunction persisting beyond one-month post-injury, initiation of vestibular and/or cervical physical therapy may occur, as determined by the physician. Vestibular therapy aims to improve motion tolerance and balance through exercises that include adaptation by retraining the vestibular ocular reflex. While some physical therapists incorporate pencil pushups or Brock string (common treatments for CI) into their vestibular therapy protocols, for this study, physical therapists will be instructed not to include any convergence exercises targeting CI during SCC.

### Data analysis

#### Statistical model

*A priori* sample size calculation was performed using paired t-tests with equal variance for TYP-CI patients from both placebo/sham therapy and OBVAT. The CITT defined the clinically relevant true mean difference for CISS, NPC, and PFV as 10 points, 4 cm, and 10Δ with a standard deviation of 12, 4.5, and 11.3, respectively. The previous pilot data had an estimated correlation of -0.3, 0.9, -0.4, and -0.3 for CISS, NPC, PFV, and beta weight from oculomotor vermis for functional, respectively [81]. Hence, the resultant standard deviation of the difference (OBVAT–placebo/sham therapy) is 19, 2, 19, and 27. Assuming 80% power, Alpha = 0.05, and adjusting for an 80% retention rate and 15% data loss due to motion artifacts during fMRI experiments results in the number of participants needed to be 37, 6, 37, 46, respectively. Using the maximum sample size for all conditions to be satisfied yields a group size of 46 per arm. To round up, the number of participants for a statistically powered study would be 50 CONC-CI for each arm (i.e., 50 CONC-CI into OBVAM+SCC and 50 CONC-CI into SCC only for six weeks).

In the unlikely event that the sample size power analysis is not valid during post hoc analyses computation, an additional 20 participants will be recruited where each group would be about 60 participants for a total of 120 total participants. This group size is substantially greater than the 25-participant arm size in other typical convergence insufficiency randomized clinical trials assessing the same primary measures of optometric signs and symptoms used within this current study [82]. Hence, it is highly likely that the proposed 50-participant arm size will be sufficient; however, the contingency is to increase the arm size to 60 participants if needed or 120 total participants. Furthermore, if the post hoc test supports insufficient statistical power, then methods to decrease variance within the dataset will be applied. The following methods will be explored for the objective assessment measurements. If for the imaging analyses, the power analysis is not sufficient then rather than performing whole-brain analyses, a region of interest (ROI) based analysis will be employed. If power analysis is insufficient for eye movement analyses, then additional filtering of eye movement data will be employed, and further averaging will be done.

The CONCUSS randomized clinical trial’s primary aim is to investigate the longitudinal changes for the immediate compared to delayed OBVAM arms. To assess the effects of time, group, and their interaction on clinical measures, a mixed model analysis will be employed [83]. This model will include fixed effects for Time (baseline, outcome 1, outcome 2), Group (immediate OBVAM therapy, delayed OBVAM therapy), and their interaction (Time × Group), with random intercepts for participants to account for the repeated measures within participants. The formula for the mixed model is the following:

Dep ~ 1 + Time + Group + Time:Group + (1 | Participants)

Where:

- Dep is the dependent variable for PFV, NPC, CISS, and secondary measures including imaging parameters, metrics for eye movement assessments, other clinical signs, and additional participant symptom surveys.
- Time is an independent variable for baseline, outcome 1, and outcome 2.
- Group is an independent variable for immediate OBVAM treatment and delayed OBVAM treatment.
- Time: Group is the interaction between Time measurements and Group measurements.
- (1 | Participants) is the random intercept accounting for within-participant variability.

The mixed effects model accounts for the correlation between repeated measures by including random intercepts for participants. This approach models within-participant correlation appropriately. Interaction effects between Time and Group will be examined, and post-hoc tests will be conducted to explore the within-group and between-group differences over time. Bonferroni correction will be applied to post-hoc comparisons to control for Type I errors. Since a mixed model will be employed, the normality of the residuals of the mixed-effects model after accounting for fixed and random effects will be checked [84]. Standard checks to assess the assumptions of the mixed-effects model will be conducted. These include checking the residuals’ normality via diagnostic tools such as the Shapiro-Wilks test, Q-Q plots, and residual distribution histograms. These steps will ensure the robustness of our model’s assumptions. However, if the residuals continue to deviate significantly, then a generalized linear mixed model (GLMM) to account for non-normal distributions will be employed.

For the secondary analysis, the same mixed-effects model framework as applied in the analysis for the primary outcomes for the secondary outcomes will be used. Specifically, models will be fit to account for fixed effects of time, group, and their interaction (Time × Group), with random intercepts for participants. Post-hoc tests will be performed to further explore the main effects and interactions. Subgroup analyses to explore whether the effects of time and group differ by key demographic or clinical variables, such as age and sex will be conducted. For these analyses, the mixed-effects models including interactions between the subgroup variables and the fixed effects (e.g., Time × Group × Sex or Time × Group × Age) will be performed. These analyses will allow the investigation of whether the observed effects are moderated by specific participant characteristics. For all exploratory secondary analyses, additional covariates, such as time since injury, number of concussions, mechanism of injury, sex, and age to understand the impact of these covariates on the outcome measures will be investigated. In addition, for imaging statistical analyses, average framewise displacements will be included as a participant-level covariate. Analyses will explore whether including these covariates in the mixed-effects models changes the primary results. Non-linear models will be considered if the relationship between predictors and outcomes appears to deviate from linearity.

#### Objective eye movement data analysis

Eye movement data will first be calibrated from voltage values to degrees of rotation. Vergence eye movement data will be filtered with a 4^th^-order Butterworth low pass filter with a cutoff frequency of 40 Hz, and saccades will be filtered using a cutoff frequency of 100 Hz. Blinks are easily identified because the signal will saturate, and eye movement responses that blink during the transient or dynamically moving portion of the eye movement trace will be omitted. Latency will be assessed as the time when the eye moves towards the new visual target quantified as a 5% change in the intended vergence demand. Peak vergence velocity will be computed by taking the difference in the left and right eye movement traces and applying a two-point central difference algorithm to compute the derivative of the position trace. Convergence will be plotted as positive and divergence as negative. Peak saccadic velocity will be calculated by averaging the left and right eye movement traces and applying a two-point central difference algorithm to compute the derivative. The number of saccades, the magnitude of saccades, and whether the saccade(s) generated more or less error in attaining the final vergence position will be assessed as done in prior research [85].

Rightward saccadic movements will be positive, and leftward will be negative. The final amplitude will be the average of the last 0.5 seconds of the positional traces, where blinks will be omitted from the trace. For vergence steps and saccades, latency, peak velocity, and final amplitude will be assessed for all the eye movement response types. The absolute difference between the stimulus target and oculomotor response will be evaluated for vergence ramps to determine how much error is present for slow (1⁰/sec) and fast (4⁰/sec) convergence and divergence ramps. For the endurance analysis, the peak velocity of the 4⁰ convergence steps will be assessed during the battery and the endurance tests as a function of experimental time. A linear regression will be performed for all the responses where a downward linear slope will show a decrease in performance, a flat line will show no substantial change in performance, and an upward line slope will show a performance improvement. This analysis will be repeated for divergence and saccadic eye movements.

#### Advanced imaging data analysis

The following toolboxes will be used to analyze the imaging datasets: Statistical Parametric Mapping (SPM) (https://www.fil.ion.ucl.ac.uk/spm/software/) in MATLAB, FMRIB’s Software Library (FSL) [86] (https://fsl.fmrib.ox.ac.uk/fsl/fslwiki/) and Analysis of Functional Neuroimages toolbox (AFNI) [87] (http://afni.nimh.nih.gov/afni/). For each of the software packages, the same software version for each data processing platform will be maintained throughout the study analysis. The first five time points will be removed for each fMRI dataset to reduce T1-relaxation effects. A general linear model (GLM)—based activation analysis will be performed for the stimulus-induced tasks to derive participant-specific functional activation maps. Group-level analyses will be performed to study differences between the task-induced functional activity in both arms between Outcome 1 and between the enrollment and Outcome 2 measurements. Functional connectivity methods, including Independent Component Analysis (ICA) [88], Region of Interest (ROI) analysis, seed-to-seed and seed-to-voxel approaches [89], Amplitude of Low-Frequency Fluctuations (ALFF) [90], and regions of Homogeneity (ReHO) [91], will be performed on the rs-fMRI dataset. For the ASL data analysis, statistical models will be performed to investigate cerebral blood flow (CBF) changes and how cerebral perfusion changes in response to different interventions in CONC-CI participants. Correction for multiple comparisons will be used at *p*<0.05. A mixed model analysis will be used to study the time by group interaction with the statistical package R using the Jamovi Software [92]. The significance level will be set at *p* < 0.05.

Finally, the objective is to implement robust statistical methods that can elucidate potential connections between brain physiology and oculomotor function during the task and rest and in CONC-CI participants. This analytical approach will enable us to investigate disparities in the effectiveness of immediate OBVAM compared to delayed OBVAM while also exploring the influence of time and dosage on this population. To this end, comprehensive correlation analysis will be performed between all our measurements, including the clinical measures, oculomotor test, and task-induced and rsfMRI scans. For significant results in the group-level fMRI analysis, functional brain activation (beta weights) and other functional brain measures will be extracted for each participant. These functional brain measures will be used in a linear regression framework as dependent variables, along with clinical and oculomotor measures as independent factors to quantify the relationship between brain physiology and oculomotor function in CONC-CI participants and the impact of OBVAM on these relationships.

#### Data management plans

A database will be set up for all collected data from each participant. Data related to each participant’s name would be coded by participant ID. Matched names and participant IDs will only be shared with the research coordinator and Project Principal Investigator (TLA). All collected data will be visually checked for data quality. Participants in both treatment groups will be monitored weekly during the treatment sessions and follow-up visits for any adverse event(s) or unanticipated problem(s). The Office for Human Research Protections (OHRP) definitions of a serious adverse event, unexpected event, definitely-related, probably-related, possibly-related, and unrelated will be used [93]. Upon the occurrence of an unanticipated problem, the Adverse Event Form will be completed by the Principal Investigator (PI) and faxed to the IRB within 7 days. The PI will also inform participants of any important new information that might affect their willingness to continue participating in the research. If an adverse event necessitates changes to the consent/assent form(s) and/or protocol, that notification will be given to currently or previously enrolled participants, an amendment request will be submitted in conjunction with the adverse event report. The IRB will decide whether any new findings, new knowledge, or adverse events should be communicated to participants.

The research team will thoroughly discuss all discrepancies to achieve a group consensus. In cases where necessary, consultations with experts in clinical trials will be pursued for additional insights. Imaging data will be uploaded to NIH CDE https://cde.nlm.nih.gov/home and NIH FITBIR (Federal Interagency Traumatic Brain Injury Research Informatics System) (https://fitbir.nih.gov) using the Brain Imaging Data Structure (BIDS) format (neuroimaging.io) with the clinical signs, symptoms, and eye movements raw data and summary metrics.

#### Ethical considerations

This study is registered at ClinicalTrials.gov (Identifier: NCT05262361; March 2, 2022, https://clinicaltrials.gov/study/NCT05262361). The protocol of this study is approved by the ethical committee of the New Jersey Institute of Technology, Rutgers University-Newark, Children’s Hospital of Philadelphia, and Salus University. An alliance agreement was reached between institutions using the SMART IRB platform. NJIT is the IRB of record. Annual progress reports are required. All participants or their legal guardians will give written informed consent before enrolling in the study, and written assent will be required if the participant is a child. The nature and all steps of the study will be explained to the participants and their legal guardians, if the participant is a minor, before giving consent to the study inclusion, and all their questions will be addressed during the study. All participants will be made aware that they can withdraw from the study at any time of the study without any consequences and will still have care administered by their principal physician. The matched participant IDs and identifiable information of all participants will not be shared with the research team other than the research coordinators and the Project Principal Investigator (TLA). An online database and survey platform (REDCap) will be used during the clinical data collection and treatment sessions to record all the dataset inputs (redcap.research.chop.edu). All oculomotor and imaging data will be anonymized and only have participant IDs. Authorized research team members who need to access the data for further analysis will only have access to the anonymized final datasets. Due to the non-invasive nature of all assessments and interventions, health risks are not anticipated during the study using objective measurements beyond the provocation of concussive symptoms that the participant is already experiencing.

#### Status of study

The NIH funding was received on September 1, 2021. Recruitment began on February 21, 2022, for both NJIT and CHOP clinical sites and is estimated to continue until September 1, 2025. The project is planned to be completed on August 28, 2026. Instrumentation has been installed [94] and all study personnel have been trained and certified. Vision therapists will be recertified annually to ensure a consistent administration of OBVAM.

## Discussion

### Importance of CONCUSS RCT

The scientific rigor of CONCUSS is built on the foundation of prior RCTs, specifically, CITT, CITT-ART, and CINAPS, to generate unbiased knowledge of the visual neural mechanism of CONC-CI and how OBVAM may change the underlying neural substrates to potentially lead to an improvement in optometric signs, symptoms, and oculomotor function. Ultimately, CONCUSS will provide evidence to support best practices to aid clinicians in personalized point-of-care treatment of CONC-CI. This knowledge will impact the lives of adolescents and young adults with CONC-CI by comparing two treatment approaches (immediate OBVAM compared to delayed OBVAM), the impact of ’watchful waiting,’ and dosing differences (16 versus 12 one-hour OBVAM sessions). With this knowledge, physicians will be positioned to develop more effective diagnostic and therapeutic strategies for treating CONC-CI, which can lead to a faster return to school, sports, or work. Once the underlying neuro-mechanism of OBVAM for CONC-CI is better understood, treatment success rates and duration can be optimized, potentially leading to a lower economic burden of concussion for patients.

The novel innovation of CONCUSS is to understand the neuro-mechanism of OBVAM for CONC-CI with objective movement recordings and multimodal functional brain imaging correlated with conventional clinical signs and symptoms. There are some limitations to CONCUSS. Objective-based methods to better understand the accommodation and vestibular systems would strengthen the study; however, given how symptomatic participants with CONC-CI can be, longer assessments would not be practical.

#### Dissemination plans

The results of this study will be published in peer-reviewed scientific journals. Current models describing vergence [95] will be modified to consider the impact of concussion on the convergence system. The study findings will also be disseminated at major national and international scientific conferences. This study’s findings can be broadly disseminated to test the effectiveness of other therapies in visual neuroscience, studying motor learning and rehabilitation (applications ranging from basic science to neurological injury/disease) in conditions such as neurodegenerative diseases. The results of the objective and clinical evaluations will be provided in an easy-to-understand report format for all participants who would like to receive their results. OBVAM will be taught in interactive workshops at the American Academy of Optometry and other clinical professional societies.

#### Amendments to the study

The PI (TLA) and the lead clinicians (MS and AG) will discuss any study amendments. If an unexpected change is needed, an amendment will be submitted to the IRB, and the protocol will be revised in clinicaltrials.gov. Since all participants will be under the care of a physician, any unanticipated symptoms or events will be discussed in consultation with the participant’s physician. If any subsequent injuries occur while a participant is undergoing OBVAM, then the physician will decide whether OBVAM needs to be suspended or reduced from the dosage of twice per week. The physician will be responsible for terminating treatment if any complications arise, and the IRB will be notified.

#### Risk management

OBVAM is considered a low-risk therapeutic intervention. If a participant begins to show regression of progress or other complications arise, the primary physician will be consulted immediately to determine the next steps of treatment. The primary physician has the right to withdraw any participant from the trial if unexpected worsening of symptoms occurs. After a participant finishes the 16 sessions of OBVAM, if they have not remediated the primary care physician will determine what type of other therapeutic intervention(s) including additional vision therapy sessions may be prescribed to further improve function and reduce symptoms.

## Supporting information

S1 Checklist SPIRIT checklist. (DOCX)

S1 Appendix The manual of procedure for office-based vergence accommodative with movement therapy and clinical optometric exam procedures is located here: https://centers.njit.edu/vision/. (PDF)

S1 File Consent form. (PDF)

S2 File TrialStudyProtocol. (PDF)
