# Supplementary material for: Effectiveness of treatment for concussion-related convergence insufficiency: The CONCUSS study protocol for a randomized clinical trial
Source: PLoS One. 2024 Nov 15;19(11):e0314027. doi: 10.1371/journal.pone.0314027 (PMC11567536; doi:10.1371/journal.pone.0314027)
Supplement: S1 Appendix — (PDF) [file pone.0314027.s002.pdf]

Manual of Procedures for Office-Based Vergence Accommodative and Movement Therapy (OBVAM) as a therapeutic intervention for patients with concussion-related convergence insufficiency (CONC-CI)

OBVAM is assessed as part of the CONCUSS randomized clinical trial, supported by the National Eye Institute of the National Institutes of Health NEI R01EY023261 to Tara L Alvarez, PhD (alvarez@njit.edu) Study PI

OBVAM is designed and written by Mitchell Scheiman, OD PhD (mscheiman@salus.edu)

OBVAM is indexed via https://clinicaltrials.gov/study/NCT05262361

OBVAM with Home Reinforcement

1.1. General Principles and Guidelines for OBVAM 2
1.1.1. Therapist Instructions 2
1.1.2. Participant Instructions 7
1.1.3. Weekly Office Visits 7
1.1.4. Investigator Instructions 7
1.1.5 Treatment Compliance 7
1.2. OBVAM Vision Therapy Equipment Needed 8
1.3. Phase 1 Office Therapy Procedures List 9
1.3.1. Brock String (Level 1) 10
1.3.2. Brock String (Level 2) 12
1.3.3. 3 Dot Card 13
1.3.4. Vectograms (Quoits) Base-out 14
1.3.5. VTS4: Computer Orthoptics Random Dot Stereogram (RDS) Base-out 16
1.3.6. Loose Lens Accommodative Rock (Level 1) 17
1.3.1. Letter Chart Accommodative Rock (Level 1) 18
1.3.8. Bulls Eye Rock (Level 1) 19
1.3.9. Lens Sorting (Level 1) 20
1.4. Phase 2 Office Therapy Procedures List 22
1.4.1. Vectograms (Quoits/Clown) 23
1.4.2. VTS4/Computer Orthoptics Random Dot Stereogram (RDS) 26
1.4.3. Life Saver Cards 27
1.4.4. Loose Lens Accommodative Rock (Level 2) 28
1.4.5. Letter Chart Accommodative Rock (Level 2) 29
1.4.6. Bulls Eye Rock (Level 2) 30
1.4.1. Lens Sorting (Level 2) 31
1.5. Phase 3 Office Therapy Procedures List 33
1.5.1. Vectograms (Quoits/Clown) Jump Vergence 34
1.5.2. VTS4/Computer Orthoptics Random Dot Stereogram (RDS) (Phase 3) 36
1.5.3. Aperture Rule 37
1.5.4. Eccentric Circles 38
1.5.5. Stereoscope Bi-Ocular Rock 40
1.5.6. Prism Dissociation Bi-Ocular Rock 41
1.5.1. Computer Orthopter Bi-Ocular Rock 43
1.6. Phase 4 Office Therapy Procedures List 44
1.6.1. Compuer Orthoptics (RDS) Jump Vergence (Phase 4) 45
1.6.2. Aperture Rule Jump Vergence 46
1.6.3. Eccentric Circles Jump Vergence 47
1.6.4. Polaroid Bar Reader/Flippers 48
1.6.5. Aperture Rule/Flippers 49
1.1. Home Therapy Equipment and Sequence 50
1.1.1. Brock String (Level 1) 52

1.1.2. Brock String (Level 2) ... 53
1.1.3. 3 Dot Card... 54
1.1.4. Voluntary Convergence ... 55
1.1.5. Home Therapy System (HTS) (Level 1) ... 56
1.1.6. Letter Chart Accommodative Rock (Level 1) ... 57
1.1.1. Life Saver Cards ... 58
1.1.8. Home Therapy System (HTS) (Level 2) ... 59
1.1.9. Home Therapy System (HTS) (Level 3) ... 60
1.1.10. Eccentric Circle ... 61
1.1.11. Eccentric Circle Jump Vergence ... 63
1.1.12. Polaroid Bar Reader/Flippers ... 64
1.8 Maintenance Therapy ... 65
Appendix: Vision Treatment Protocol Summary Form ... 67

Office-based Vergence/Accommodative Therapy with Movement and Home Reinforcement (OBVAM)

1.1 General Principles and Guidelines for OBVAM

OBVAM requires a participant to undergo a specific therapy regimen with 16 weekly, 60-minute in-office treatment sessions. Vision Therapists administer the therapy in the office. OBVAM procedures are then supplemented with various home therapy procedures.

1. The OBVAM program has been divided into 4 phases (See Appendix). Within each phase there are a number of categories such as concussion-related saccades, concussion-related pursuits, gross convergence, vergence, and accommodation.
2. The therapy procedures in each category have been arranged sequentially from easiest to most difficult.
3. Each therapy procedure is described in this manual. The description includes the objectives, required equipment, procedure setup, procedure description, and the endpoint.
4. The endpoints are reasonable estimates of what we believe each participant should be able to achieve. These endpoints should be considered guidelines, rather than rigid criteria. Thus, if a participant appears to have attained the stated objectives of the therapy technique but is unable to achieve the precise endpoint, the therapist may move to the next procedure after a reasonable effort. The therapist should continue to work on prior techniques to achieve endpoints.
5. We anticipate that approximately 6 visits will be required to complete phase 1, 4 visits for phase 2, and 3 visits for phases 3 and 4. This is only a guideline and in some cases a participant may complete the techniques in one phase sooner or later than this estimate. There is no minimum amount of time required for each phase. The therapist can move from one phase to another as quickly as the participant reaches the stated endpoints.
6. It is possible for a participant to complete the entire OBVAM sequence in less than 16 weeks. If this occurs, the participant will continue to come in and the therapy procedures used for these visits will be the final procedures (most difficult) in each category (gross convergence, vergence, and accommodation).
7. The principal therapist at each site will be responsible for monitoring the progress with therapy and recommending changes to the procedures performed at each visit as needed.
8. The vision therapy will always be performed on a one to one basis (one therapist per participant).

1.1.1 Therapist Instructions

It is important to understand that there are general principles and guidelines that apply to all binocular vision and accommodative techniques. Vision therapy is similar in many ways to other types of therapy that involve learning and education. If we look at other types of learning it becomes clear that there are specific guidelines to facilitate learning and success. Since vision therapy can be considered to be a form of learning and education, similar principles and guidelines are used to achieve success. Therapists should adhere to the following guidelines:

Determine a level at which the participant can perform easily

Working on this level makes it easier for the participant to become aware of the important feedback cues, strategies, and objectives involved in vision therapy. It also builds confidence and motivation.

Be aware of frustration level

Signs of frustration include: general nervous and muscular tension, hesitating performance, and possibly a desire to avoid the task.

Use positive reinforcement
The participant should be rewarded for attempting a task, even if it is not successfully completed. Reinforcements can be verbal praise, tokens that can be exchanged for prizes, or participating in a task that the participant enjoys.

Maintain an effective training level
Start at the initial level at which the task is easy and gradually increase the level of difficulty, being very careful to watch for signs of frustration. Vision therapy should be success-oriented, that is, build on what the participant can do successfully as opposed to giving tasks that are too difficult.

Emphasize to the participant that changes must occur within his or her/her own visual system
A key to success in vision therapy is teaching the participant to internalize changes in visual function, as opposed to just achieving certain criteria for specific techniques. Often, as participants go through a vision therapy program, they gain the impression that it is the instrumentation, lenses, or prisms that affect the change in their visual system. Unless told otherwise, a participant may believe that these external items are the keys to their success in vision therapy.

It is important to stress that the participant must be made aware that the changes actually occur internally, within the visual system, and not externally in the instruments and paraphernalia utilized in vision therapy. To accomplish this objective the language used in communication between the therapist and participant is critical.

Examples:
When performing a fusional vergence technique the therapist might say

"Try and keep the picture single."

The problem with this instructional set is that while the participant is asked to try, the instructions are given in terms of what happens to the targets rather than what changes the participant must make internally to achieve the desired result.

Try the following approach instead.

Explain to the participant that if the picture is double it is because he or she is looking too far or too close in space. In order to make it single he or she needs to look nearer or farther; he or she needs to make adjustments within him or herself, in where he or she is pointing his or her eyes in space, and then the picture will become single.

The underlying important concept is that it is not just the specific technique that leads to success in vision therapy. Rather, the key factor is to get the participant to take responsibility for creating internal change.

Make the participant aware of the goals of vision therapy
The participant must know why he or she is in vision therapy. He or she should be able to explain what his or her problem is, how it affects performance and the goals of vision therapy. Even with a young child, the therapist should try to establish some understanding on the part of the child about what is wrong with his or her eyes and why vision therapy is necessary. For each therapy technique the child should be able to explain what he or she needs to do to accomplish the desired task.

Set realistic therapy objectives and maintain flexibility with these objectives or endpoints
With all therapy techniques there are certain general objectives that we expect to achieve before we proceed to the next procedure. Each technique in this manual has specific "endpoints." It is important to understand that these endpoints are only guidelines and that flexibility and clinical judgment are important in deciding when to move on to another procedure. The objective of vision therapy is to achieve the objectives of the technique as quickly as possible. If a participant can only achieve 23 Base-out (endpoint 25 Base-out) with the Quoits/Clown Vectogram procedure in spite of sufficient effort then it makes sense to move on and try another technique.

Use vision therapy techniques that provide feedback to the participant
When performance feedback is available to the participant, therapy and teaching progresses more effectively. The various feedback mechanisms used in vision therapy include:

1. Diplopia
2. Blur
3. Suppression
4. Luster
5. Kinesthetic awareness
6. SILO (Small In, Large Out) (use of vergence as a cue for distance perception)
1. Float
8. Localization

Methods to Overcome Obstacles and to Facilitate Progress in OBVAM
If a participant is experiencing difficulty converging, diverging, relaxing, or stimulating accommodation, it is important to try and help the participant overcome this obstacle, rather than simply make the task easier. The following techniques should be used throughout the therapy program to accomplish this objective.

Participant is Experiencing Difficulty with Gross Convergence

1. Suggest that participant try and get the “feeling” of looking close and crossing his or her eyes.
2. Have the participant touch the object that he or she is trying to fuse. This kinesthetic feedback is sometimes enough to help the participant achieve single vision.
3. Use binocular minus lenses to stimulate accommodative convergence.

Participant is Experiencing Difficulty with Positive Fusional Vergence

1. Suggest that the participant get the feeling of looking close and crossing his or her eyes.
2. Use the following feedback technique of localization to show him how to regain fusion.

Localization refers to the ability of the participant to point to where the target appears to be when fusion occurs, and is based on the concept of physiological diplopia. During a convergence technique the visual axes cross before the target and the participant should perceive the target as smaller and

closer. The participant can now be asked to pick up a pointer and point to where he or she sees the target "floating." The objective is for the participant to point to the target and perceive one target and one pointer.

If the participant places the pointer in the general area of where his or her visual axes cross, he or she will perceive one target and one pointer. If he or she points closer or farther away than the intersection of his or her visual axes, he or she will report diplopia of either the target or the pointer. The importance of localization is that it allows the participant to develop an understanding of what changes must occur within his or her visual system to accomplish the therapy task. If he or she can localize the target, he or she will begin to understand that when the targets are separated to create a convergence demand, he or she must look closer and cross his or her eyes to maintain single vision and fusion. We cannot overemphasize the importance of the participant developing this understanding of what changes he/she must make to accomplish a particular task.

Often when a participant is first asked to try and localize during convergence therapy, he or she experiences difficulty. At first he or she may tend to point to the actual plane of the target, rather than the intersection of the visual axes. It is useful to state:

"We both know that the targets are back there, but what I want you to do is to try and get the feeling of where you are looking and where the target is floating."

If the participant continues to have problems localizing, the next step is to make him aware of the concept of physiological diplopia and to use this phenomenon to get the participant started. The explanation we use with participants is as follows:

"The way the visual system works is that whatever object we are directly viewing is seen as one, all other objects are seen as double." It is then useful to demonstrate this by having the participant look at a pointer while you hold another object in the background. Have the participant experiment with this concept for several minutes until he or she is comfortable with this idea and is satisfied that he or she can experience physiological diplopia. Demonstrate that when the more distant object (seen as two) is moved closer to the fixation object, it will also be seen singly when it is in approximately the same position in space. If the participant now understands the concept that we experience single vision when pointing to where the eyes are looking, the idea can be applied to vision therapy techniques. For example, we are working with a Quoits/Clown Vectogram and a convergence demand and the participant when asked to localize, points too far away and experiences diplopia. If the participant understands the concept of physiological diplopia we would say the following:

"This time I want you to hold the pointer at the slides and look directly at the pointer. Do not try to keep the two Quoits/Clowns single. If you look at the pointer while you do so you will see two Quoits/Clowns in the background. Now slowly move the pointer toward you, always looking directly at the pointer and being aware of the two Quoits/Clowns. As you do this you will notice that as you move the pointer toward you, the two Quoits/Clowns appear to move closer to one another. Continue moving the pointer toward you very slowly and you will notice that at some distance you will see one pointer and one Quoits/Clown. This is where you must look to accomplish this task. Do you feel yourself looking closer? Try and get the feeling of where you have to look. Can you now understand where you have to look to see one Quoits/Clown? Can you see that the Quoits/Clown is floating closer?"

Generally the participant continues to be unable to simply pick up the pointer and immediately localize correctly. However, with repetition most participants will soon understand what they must do visually during convergence therapy.

As a last resort you may decrease the demand of the task. Rather than simply moving the targets closer together, it is preferable to use lenses or prism to accomplish this goal.

a. Minus lenses
b. Base-in prism
c. Increase the working distance

Participant is Experiencing Difficulty with Negative Fusional Vergence

1. Suggest that the participant get the feeling of looking farther away and relaxing his or her eyes.
2. Use the following feedback technique of localization to show him how to regain fusion.

   The following divergence therapy procedure is a powerful training technique and in most instances will lead to excellent progress with divergence therapy. The participant is asked to stand several feet in front of a ball that has been suspended from the ceiling. The height of this ball should be adjustable to permit the therapist to change the height so that it is at eye level for any given participant. A Quoits Vectogram is placed in a clear holder and the participant is instructed to hold the target at arm's length so that he or she can see the ball in the background directly in the center of the Quoits. As the Quoits targets are slowly separated to create a divergence demand the participant is asked to maintain fusion and describe where the target is floating. At this point the therapist pushes the ball to create motion in an arc moving towards and away from the participant. The participant should perceive that the ball is moving in front and behind the Quoits, which itself appears to be floating behind the plane of the actual Vectogram targets. As the targets are separated, the participant will have to continue moving backwards to keep the Quoits floating out at the point at which the ball just swings in front of and behind the Quoits. Once appreciated, this is quite a startling experience for the participant and provides the feedback necessary for him to understand that when fusing during divergence therapy he or she has to relax his or her eyes as if something is moving farther away from him.
3. As a last resort you may decrease the demand of the task. Rather than simply moving the targets closer together it is preferable to use lenses or prism to accomplish this goal.
   a. Plus lenses
   b. Base-out prism
   c. Increase the working distance

Participant is Experiencing Difficulty with Stimulation of Accommodation

If the participant is experiencing difficulty at any level:

1. Suggest that participant try and get the "feeling" of looking close and crossing his or her eyes.
2. Decrease the demand by moving card away until the print is clear and then moving back to 40 cm.
3. As a last resort decrease the demand by decreasing the power of the lenses.

Participant is Experiencing Difficulty with Relaxation of Accommodation

If the participant is experiencing difficulty at any level:

1. Suggest that participant try and get the "feeling" of looking farther away, relaxing or staring.
2. Decrease the demand by moving the card closer until the print clears and then move back to 40 cm.

3. As a last resort decrease the demand by decreasing the power of the lenses.

1.1.2 Participant Instructions

Participants will be given a written set of instructions which describe how to perform the home therapy procedures. The participant will be given, at each therapy visit, new home procedures to perform 15 minutes, 3 times per week. The therapist will review this instruction sheet in detail at the first treatment and at each weekly office visit to ensure that the participant has a complete understanding of the technique. Participants will be asked to demonstrate all home therapy techniques to the therapist before leaving. In addition, the participant will be given a home log form and instructions for proper completion.

1.1.3 Office Visits

The participants in this group will meet weekly with the therapist. During the 60-minute office visit, much of this time will be spent on in-office therapy procedures. However, time must also be allotted to review the home therapy procedures. The therapist will question the participant about his/her home therapy procedures during the previous week and check the participant’s home therapy log and progress. This will be an opportunity for the therapist to correct any errors in technique, provide suggestions about how to overcome any perceived obstacles with the treatment, and to encourage and motivate the participant.

The therapist should make every attempt to emphasize compliance and question the participant about problems/issues with home or office therapy. However, the therapist should not initiate discussion about the participant’s symptoms. If such issues arise, the participant can be directed to the principal therapist for further discussion. The therapist should not have access to the participant’s binder specifically results from the masked examinations. During weekly meetings between the principal therapist and therapist to review participant progress, the discussion should be directed towards progress with therapy procedures and/or protocol issues.

1.1.4 Treatment Compliance

Participants will be required to keep a daily home log of the time spent on each activity/ procedure and bring the form with them to the office appointments (Home Log Sheet).

1.2 OBVAM Vision Therapy Equipment Needed

Brock String
Dual Polachrome Orthopter/Illuminated Trainer
Quoits/Clown/Spirangle Vectograms
Loose Lenses (+2.00 D to -3.00 D in 0.50 D increments and -3 to -6 in 1.00 steps)
Accommodative Hopping cards
Hart Chart (large and small letter chart cards)
Aperture Rule and cards
Clear Eccentric Circles
Opaque Eccentric Circles
Lens Flippers (+1.00/-1.00,+1.50/-1.50, +2.00/-2.00)
Home Therapy System computer software (HTS)
VTS4 Computer Orthoptics Random Dot Stereogram (RDS) and Accommodative software
Polaroid Glasses
Eye patch
Pointer

Home Therapy Equipment

Brock String
HTS
Red and Blue glasses
Eccentric Circles

1.3 Phase 1 Office Therapy Procedures List

Concussion-related saccades

Level 1: 2 Pencils – Horizontal (head still), slow to fast
Level 2: 2 Pencils – Vertical (head still), slow to fast
Level 3: 2 Pencils – Horizontal (head still), slow to fast walking forward and backward
Level 4: 2 Pencils – Vertical (head still), slow to fast walk in circle
Level 5: 2 Pencils – Horizontal with slow to fast rotating body

Concussion-related pursuits

Level 1: Hanging Ball –Lateral arc, body still
Level 2: Hanging Ball –forward and backward arc, body still
Level 3: Hanging Ball – Lateral arc with body rocking L to R, opposite direction of ball
Level 4: Hanging Ball – Lateral arc and circle around swinging ball

Gross Convergence Techniques

Brock String (Level 1)
Brock String (Level 2)
Brock String (Level 3)

Fusional Vergence Techniques

Vectograms (Quoits/Clown)
VTS4 (RDS)

Monocular Accommodation Techniques

Loose Lens Accommodative Rock
Letter Chart Accommodative Rock

1.3.1 Concussion-related Saccades Level 1 (Horizontal – no walking)

Objective

To help you eliminate your motion sickness and vision symptoms by improving your eye comfort, speed, accuracy, and efficiency when looking from one point to another

Equipment Needed

1. 2 pencils

Setup

1. Have the participant hold one pencil in each hand at eye level about 12-16 inches away and separated by about 6-8 inches from the center.

Procedure

1. Instruct the participant to focus on the right pencil. As soon as it is clear and single, change focus to the left pencil. As quickly and accurately as possible, keep alternately looking from one pencil to the other.
3. When looking from one pencil to the other, hold the right pencil in place, and slowly move the left pencil closer towards the right pencil. Keep making saccades even while the second pencil is moving. Move it out to the original position and begin again.
4. Modify the separation and speed of moving the pencils as the participant progresses. The goal is good comfort with the eye movements.

Endpoint

1. Good comfort during the eye movements

1.3.2 Concussion-related Saccades Level 2 (Vertical – no walking)

Objective

To help you eliminate your motion sickness and vision symptoms by improving your eye comfort, speed, accuracy, and efficiency when looking from one point to another

Equipment Needed

1. 2 pencils

Setup

1. Similar to level 1, except now the pencils are positioned vertically a few inches above and below eye level.

Procedure

1. Instruct the participant to focus on the top pencil. As soon as it is clear and single, change focus to the bottom pencil. As quickly and accurately as possible, keep alternately looking from one pencil to the other.
3. When looking from one pencil to the other, hold the top pencil in place, and slowly move the bottom pencil closer towards the top pencil. Keep making saccades even while the second pencil is moving. Move it out to the original position and begin again.
4. Modify the separation and speed of moving the pencils as the participant progresses. The goal is good comfort with the eye movements.

Endpoint

1. Good comfort during the eye movements

1.3.3 Concussion-related Saccades Level 3 (Horizontal – walking)

Objective

To help you eliminate your motion sickness and vision symptoms by improving your eye comfort, speed, accuracy, and efficiency when looking from one point to another

Equipment Needed

1. 2 pencils

Setup

1. Have the participant hold one pencil in each hand at eye level about 12-16 inches away and separated by about 6-8 inches from the center.

Procedure

1. Instruct the participant to focus on the right pencil. As soon as it is clear and single, change focus to the left pencil. As quickly and accurately as possible, keep alternately looking from one pencil to the other. Now instruct the participant to walk 5- 10 steps forward while making these eye movements and then walk 5-10 steps backward.
3. When looking from one pencil to the other, hold the right pencil in place, and slowly move the left pencil closer towards the right pencil. Keep making saccades even while the second pencil is moving. Move it out to the original position and begin again. Now instruct the participant to walk 5- 10 steps forward while making these eye movements and then walk 5-10 steps backward
4. Modify the separation and speed of moving the pencils as the participant progresses. The goal is good comfort with the eye movements.

Endpoint

1. Good comfort during the eye movements

1.3.4 Concussion-related Saccades Level 4 (Vertical – walking)

Objective

To help you eliminate your motion sickness and vision symptoms by improving your eye comfort, speed, accuracy, and efficiency when looking from one point to another

Equipment Needed

1. 2 pencils

Setup

1. Similar to level 1, except now the pencils are positioned vertically a few inches above and below eye level.

Procedure

1. Instruct the participant to focus on the top pencil. As soon as it is clear and single, change focus to the bottom pencil. As quickly and accurately as possible, keep alternately looking from one pencil to the other. Now instruct the participant to walk 5- 10 steps forward while making these eye movements and then walk 5-10 steps backward
3. When looking from one pencil to the other, hold the top pencil in place, and slowly move the bottom pencil closer towards the top pencil. Keep making saccades even while the second pencil is moving. Move it out to the original position and begin again. Now instruct the participant to walk 5- 10 steps forward while making these eye movements and then walk 5-10 steps backward
4. Modify the separation and speed of moving the pencils as the participant progresses. The goal is good comfort with the eye movements.

Endpoint

1. Good comfort during the eye movements

1.3.5 Concussion-related Saccades Level 5 (Horizontal – with body rotation)

Objective

To help you eliminate your motion sickness and vision symptoms by improving your eye comfort, speed, accuracy, and efficiency when looking from one point to another

Equipment Needed

1. 2 pencils

Setup

1. Have the participant hold one pencil in each hand at eye level about 12-16 inches away and separated by about 6-8 inches from the center.

Procedure

1. Instruct the participant to focus on the right pencil. As soon as it is clear and single, change focus to the left pencil. As quickly and accurately as possible, keep alternately looking from one pencil to the other. Now instruct the participant to rotate the body (spin like a top) while making these eye movements.
3. When looking from one pencil to the other, hold the right pencil in place, and slowly move the left pencil closer towards the right pencil. Keep making saccades even while the second pencil is moving. Move it out to the original position and begin again. Now instruct the participant to rotate the body (spin like a top) while making these eye movements
4. Modify the separation and speed of moving the pencils as the participant progresses. The goal is good comfort with the eye movements.

Endpoint

1. Good comfort during the eye movements

1.3.6 Concussion-related Pursuits Level 1 (Lateral Arc – Body Still)

Objective

To help you eliminate your motion sickness and vision symptoms by improving your eye comfort, speed, accuracy, and efficiency when looking from one point to another

Equipment Needed

1. Hanging ball

Setup

1. Have the participant stand in front of a hanging ball that is about 16 inches away and slightly below eye level.

Procedure

1. Instruct the participant to focus on the ball and that you are going to move it laterally in an arc.
2. Create the arc and instruct the participant to carefully follow it and to report any discomfort.
3. Modify the arc to make it easier (smaller arc, or harder larger arc)

Endpoint

1. Good comfort during the eye movements

1.3.7 Concussion-related Pursuits Level 1 (Forward and backward Arc – Body Still)

Objective

To help you eliminate your motion sickness and vision symptoms by improving your eye comfort, speed, accuracy, and efficiency when looking from one point to another

Equipment Needed

1. Hanging ball

Setup

1. Have the participant stand in front of a hanging ball that is about 16 inches away and slightly below eye level.

Procedure

1. Instruct the participant to focus on the ball and that you are going to move it forward and backward (to and away from the eyes) in an arc.
2. Create the arc and instruct the participant to carefully follow it and to report any discomfort.
3. Modify the arc to make it easier (smaller arc, or harder larger arc)

Endpoint

1. Good comfort during the eye movements

1.3.8 Concussion-related Pursuits Level 1 (Lateral Arc – Body Rocking opposite direction of ball)

Objective

To help you eliminate your motion sickness and vision symptoms by improving your eye comfort, speed, accuracy, and efficiency when looking from one point to another

Equipment Needed

1. Hanging ball

Setup

1. Have the participant stand in front of a hanging ball that is about 16 inches away and slightly below eye level.

Procedure

1. Instruct the participant to focus on the ball and that you are going to move it laterally in an arc.
2. Create the arc and instruct the participant to carefully follow it and to report any discomfort.
3. Instruct the participant to move the body left and right in the opposite direction of the ball (Ball moves right, move body to the left) and continue to carefully following the [path of the ball.
3. Modify the arc to make it easier (smaller arc, or harder larger arc)

Endpoint

1. Good comfort during the eye movements

1.3.9 Concussion-related Pursuits Level 1 (Lateral Arc – Walk a circle around ball)

Objective

To help you eliminate your motion sickness and vision symptoms by improving your eye comfort, speed, accuracy, and efficiency when looking from one point to another

Equipment Needed

1. Hanging ball

Setup

1. Have the participant stand in front of a hanging ball that is about 16 inches away and slightly below eye level.

Procedure

1. Instruct the participant to focus on the ball and that you are going to move it laterally in an arc.
2. Create the arc and instruct the participant to carefully follow it and to report any discomfort.
3. Now instruct the participant to walk a circle around the moving ball and to continue to carefully follow the ball.
4. Modify the arc to make it easier (smaller arc, or harder larger arc)

Endpoint

1. Good comfort during the eye movements

1.3.10 Brock String (Level 1)

Objective

The objectives of Brock String are to:
1. Develop the kinesthetic awareness of converging and diverging
2. Develop the ability to voluntarily converge
3. Normalize the near point of convergence

Equipment Needed
1. Brock String
2. Lens flippers (+/-2.00 D)

Setup
1. Use two beads and about 1 m of string.
2. Instruct the participant to hold the string taut and against the bridge of his or her nose.
3. Set one bead about 24 inches (red bead) from the participant and the other about 12 inches away (green bead).

Procedure
1. Ask the participant to look at the closer bead and describe what he or she sees. Because of physiological diplopia he or she should report that he or she sees one green bead and two red beads. In addition, he or she should perceive two strings crossing at the green bead with one string extending from his or her right eye and the other appearing to extend from his or her left eye.
2. Ask the participant to fixate the far bead (red) and he or she should now report one red bead with the strings crossing at the red bead. He or she will also see two green beads.
3. It is important to explain the meaning of these observations to the participant. Use the following explanation. “We are doing this exercise to teach you how to cross your eyes. The exercise lets you know what your eyes are doing at all times. The way vision works is that wherever your eyes are pointing you have single vision. Everything else in front or behind the object you are looking at will be seen as double. Look at the green bead and you will see one green bead, two red beads behind it and a string that crosses right at the green bead and forms the letter "X". The strings should look as if they are extensions of your right and left eyes. Where you perceive the two strings cross is actually where your eyes are aimed. Thus, if you are trying to look at the green bead but the strings appear to cross farther away than the bead, this is an indication that you are looking too far away. Use this information to try and correct your eye position and look closer.”
4. If the participant experiences difficulty accomplishing any of the goals listed above there are several techniques the therapist can use to help him overcome this obstacle.
    a. Suggest that participant try and get the “feeling” of looking close and crossing his or her eyes.
    b. Have the participant touch the bead that he or she is trying to fuse. This kinesthetic feedback is sometimes enough to help the participant achieve single vision.
    c. Use binocular minus lenses to stimulate accommodative convergence.
5. Once the participant is able to fuse the near and far beads instruct him to hold fixation at the near bead for 5 seconds and then switch fixation to the far bead and hold for 5 seconds.

6. Have him repeat this three times and then move the near bead 5 cm closer while always maintaining the far bead at 60 cm.
1. Have the participant repeat the step of alternately fixating the far and near beads for 5 seconds, 3 times.
8. Continue moving the near bead closer until he or she can successfully converge to a distance of 2.5 cm from his or her nose.

Endpoint

1. The participant can successfully converge to a bead placed 2.5 cm from his or her nose.
2. The participant should be able to appreciate the different feeling and effort associated with converging and diverging.

1.3.11 Brock String (Level 2)

Objective

The objectives of Brock String are to:

1. Develop the kinesthetic awareness of converging and diverging
2. Develop the ability to voluntarily converge
3. Normalize the near point of convergence

Equipment Needed

1. Brock String
2. Lens flippers (+/-2.00 D)

Setup

1. Use about 1 m of string, one bead at 2.5 cm and the other at 1 m.
2. Instruct the participant to hold the string taut and against the bridge of his/her nose.

Procedure

1. Instruct the participant to fixate the bead at the end of the string (1 m) to try and see that the two strings cross at the bead.
2. Now have the participant very slowly fixate closer and closer until he/she is fixating at the close bead at 2.5 cm in front of his/her nose. It is important to emphasize to him/her that the change in fixation from far to near should be very gradual.
3. After the participant can converge all the way to his/her nose, reverse the process and have him gradually diverge to the end of the string. Repeat this procedure for 20 repetitions.
4. Now have the participant remove all the beads from the string. Ask the participant to slowly converge from 1 m to 2.5 cm and to always see the “X.”

Endpoint

1. The participant can voluntarily converge to a distance 2.5 cm from his or her nose.
2. The participant should be able to appreciate the different feeling and effort associated with converging and diverging.

1.3.12 Brock String Level 3

Objective

Develop the ability of your eyes to voluntarily converge

Equipment Needed

1. None

Setup

No setup required

Procedure

1. You should be able to voluntarily cross your eyes without the aid of a pointer, finger or any other object. To do so you should try and imagine an object slowly moving towards your eyes and try to get the feeling of crossing your eyes like when you worked with the Brock string.
2. Once you can cross your eyes so that you are looking as close as 1-2 inches from your nose, reverse the process and gradually relax your eyes and look at a distance target. Repeat this procedure for 20 repetitions.

Endpoint

1. You should be able to voluntarily cross your eyes to a distance 2.5 cm (I inch) from your nose.
2. You should be able to appreciate the different feeling and effort associated with crossing and relaxing your eyes.

1.3.13 Vectograms (Quoits/Clown) Base-out and Base-in

Objective

To increase positive and negative fusional amplitudes

Equipment Needed

1. Quoits and Clown Vectogram
2. Dual Polachrome Illuminated Trainer
3. Polaroid Glasses

Setup

The participant wears Polaroid glasses and the Quoits Vectogram targets are set up in the Dual Polachrome Illuminated Trainer with the targets set at zero prismatic demand.

Procedure

Level 1: Establishing Basic Fusion Ability

1. Ask the participant to describe what he or she sees. The participant should be able to describe the picture and indicate that parts of the picture appear to be floating closer than other parts.
2. The participant should also see the boxes with an “R” aligned over an “L”.
3. If the participant doesn't voluntarily respond with these answers, ask leading questions to elicit this information. Once you are able to elicit these responses proceed to step two.

Level 2: Establishing Presence of Feedback Cues

A. Blur

1. Determine if the participant is able to appreciate blur by slowly increasing the convergence demand until the participant loses clarity.
2. Decrease the convergence demand until the participant regains clear vision.

B. Diplopia

1. Determine if the participant is able to appreciate diplopia by slowly increasing the convergence demand until the participant loses fusion.
2. Decrease the convergence demand until the participant regains single, clear vision.

C. SILO (Small In Large Out)

1. Tell the participant to ignore “R” and “L” initially and to concentrate on the picture.
2. While slowly separating the two sheets to create a small amount of convergence demand, ask the participant to try and keep the picture clear and single and describe what he or she is seeing. The participant should notice that the target becomes smaller and moves closer.
3. While slowly separating the two sheets to create a small amount of divergence demand, ask the participant to try and keep the picture clear and single and describe what he or she is seeing. The participant should notice that the target becomes larger and moves farther away.
4. If the participant is unable to spontaneously describe this, it is important to ask leading questions to obtain these responses.

Sample questions are:

Is the picture becoming larger or smaller?

Is the picture coming closer or moving farther away?

5. Establish whether the participant is experiencing SILO [small and in (SI) with convergence and large and out with divergence (LO)] or SOLI [small and out (SO) with convergence and large and in with divergence (LI)].

D. Float/Localization

1. After establishing that the participant appreciates SILO, slowly increase the convergence demand and ask the participant to point to where the target appears to be floating in space. Ask the participant to point to different parts of the stimulus. Explain to the participant that these are all feedback cues (blur, diplopia, SILO, float/localization) and will be used throughout therapy to help monitor his or her responses.

Level 3: Convergence Therapy

Set the targets at zero prism demand and explain to the participant that you are going to demonstrate the procedure that he or she will practice. It involves 2 distinct steps.

1. **Step One:** Tell the participant to separate the sheets to 3^{Δ} Base-out and try to maintain clear, single vision (For convergence, separate the targets so that the numbers are shown).
2. **Step Two:** Instruct the participant to take a pointer and point to the location at which he or she sees the stimulus floating. Make sure the participant sees one pointer and one target. Stress to the participant the importance of the kinesthetic awareness or feeling of "looking close" and "crossing his or her eyes."
3. Once the participant can perform these steps, while the Vectograms slides are set at 3^{Δ} Base-out, have the participant slowly separate the targets to 6^{Δ} Base-out and repeat steps 1-2. At some level the participant will be unable to successfully complete even step one, to see the circles clearly and singly.
3. If the participant is experiencing difficulty:
   a. Suggest that the participant get the feeling of looking close and crossing his or her eyes.
   b. Use the feedback technique of localization to show him how to regain fusion.

5. Once the participant can achieve clear, single vision with the Quoits Vectogram at 30 Base-out, repeat the same procedure with the Clown Vectograms.

Level 4: Divergence Therapy

This step does require a ball hanging from the ceiling placed at eye level.

Have the participant stand up and hold the Quoit Vectogram in a horizontal Vectogram holder with arms comfortably held out front and holding the Quoit right at the plane of the hanging ball. Set the targets at zero prism demand and explain to the participant that you are going to demonstrate the procedure that he or she will practice. It involves 2 distinct steps.

1. **Step One:** Ask the participant what he/she sees at that moment. They should see one clear Quoit touching the ball. Then separate the sheets to 3^{Δ} Base-in and ask the participant to maintain clear, single vision while they continue to look at the ball, but the key is that they need to move backward so that the Quoit appears to be floating in space at the plane of the ball.
2. **Step two:** Now create a forward/backward arc of the ball and the participant should now see the ball appears to move behind and in front of the plane of the Quoit. Emphasize that they should have the feeling of looking in the distance, or relaxing the eyes.

2. **Step Three:** Continue separating the Quoit by 2-3 prism diopters and have the participant move away from the ball as necessary to see the plane of the Quit to be at the position of the ball. Instruct the participant to take a pointer and point to the location at which he or she sees the stimulus floating. Make sure the participant always sees one ball and one target. Stress to the participant the importance of the kinesthetic awareness or feeling of "looking far" and "relaxing or diverging his or her eyes."
3. If the participant is experiencing difficulty:
   a. Suggest that the participant get the feeling of looking far and relaxing his or her eyes.
   b. Try the use of plus lenses.

Endpoint

30$^{\Delta}$ Base-out/12 base-in

1.3.14 VTS4: (RDS) Base-out Only

Objective

1. To increase positive fusional amplitudes

Equipment Needed

VTS4

Setup

1. Turn on controller.
2. Turn on the liquid crystal glasses. The participant wears liquid crystal glasses. (Please note that these need to be charged.)
3. Turn on monitor and click on VTS4
4. Click on continue
5. Click on Start program monitor (see figure below)
6. Click Start Program and adjust the viewing distance if needed (typical distance is 30”).
7. Confirm that the LCD shutter glasses are working properly
8. Click on Main Menu
9. At the main Menu select **“Multiple Choice Vergence”**
10. On the left hand side under “DIRECTION select **“horizontal” and “base out”**
11. On the right hand side of the screen select **“RDS”**
12. The others settings that you will see on the bottom of the screen do not require any adjustment.
13. Finally select the stimulus option with the “**blue box and the letter A**”.
14. The procedure will begin and last for 5 minutes
15. REPEAT FOR Base-in therapy
16. At the main Menu select **“Multiple Choice Vergence”**

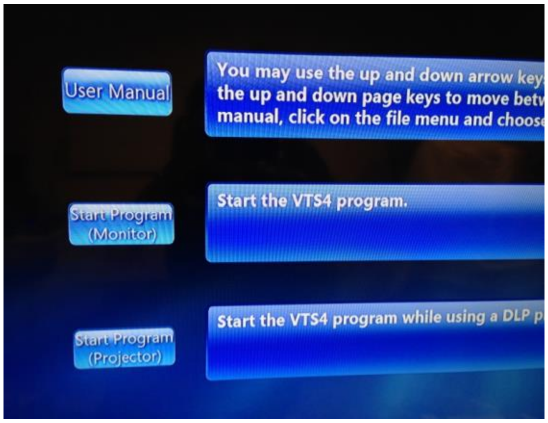

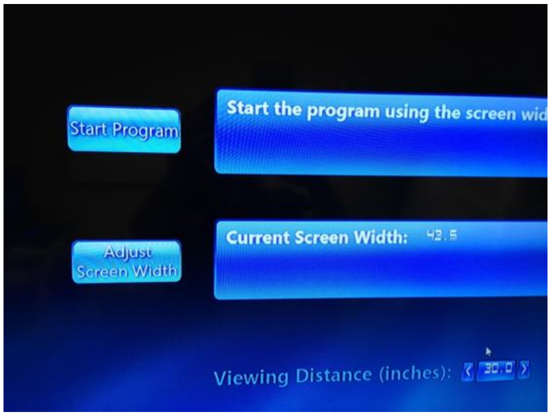

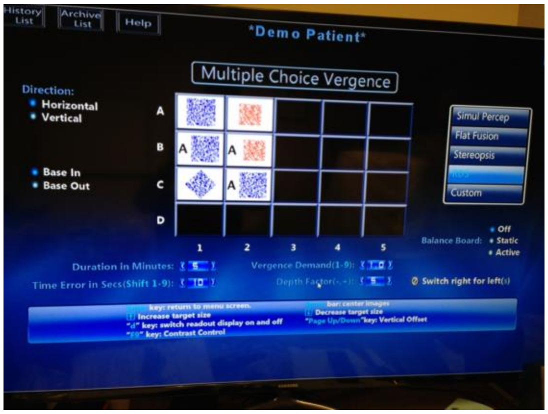

Procedure

1. Ask the participant if he or she is able to see the Letter “A” in the middle of the large square and another smaller square to be floating closer than the large square. This smaller square will either be above, below, to the right, or to the left of the letter “A”.
2. The participant should be able to move the game pad button in the direction he or she sees the floating square (right, left, up down).
3. The software program increases the prismatic demand of the task after two correct responses and decreases the prismatic demand after an incorrect response. The participant will receive auditory feedback for either a correct or an incorrect response.

Endpoint

30^{Δ} Base-out with RDS targets

1.3.15 Loose Lens Accommodative Rock (Level 1)

Objective
1. Restore normal monocular accommodative amplitude.
2. Restore normal monocular accommodative facility.

Equipment Needed
1. Hopping Accommodative Facility VT Cards
2. Loose lenses from +1.50 D to -3.00 D in 0.50 D increments
3. Eye patch

Setup
1. Occlude the participant's left eye.
2. Hopping Card (20/30) is held at 40 cm.
3. The participant is asked to hold two lenses, one in the right hand and one in his or her left hand.
4. Start with +0.50 D and –0.50 D.

Procedure
1. The participant is asked to clear the print as he/she alternately holds the plus and then the minus lens in front of his/her right eye.
2. Give the participant as much time as necessary for him/her to clear and read the print.
3. The goal is to achieve clear vision, 10 times, without regard to the time factor.
4. Increase the power of the lenses in 0.50 D increments up to the endpoints (+1.50/-3.00).
5. If the participant is experiencing difficulty at any level:
    a. Suggest that participant try and get the "feeling" of looking close and crossing his/ her eyes for the minus lenses or try and get the feeling of relaxing or staring for the plus lenses.
    b. Decrease the demand by moving card away until the print is clear and then moving back to 40 cm for the minus lenses or move the card closer until the print clears and then move back to 40 cm for the plus lenses.
    c. Decrease the demand by decreasing the power of the lenses for either plus or minus.
6. Repeat the procedure with the right eye occluded.
7. Once the participant can achieve clarity through +1.50/-3.00 lenses, begin to work on speed. The goal is to achieve 10 cpm with +1.50/-3.00.

Endpoint
1. Clear +1.50/-3.00, 10 cycles in one minute.
2. The participant should be able to appreciate the different feeling and effort associated with clearing the print through minus and plus lenses.

1.3.16 Letter Chart Accommodative Rock (Level 1)

Objective

1. Restore normal monocular accommodative amplitude.
2. Restore normal monocular accommodative facility.

Equipment Needed

1. Large Hart Chart suitable for distance viewing
2. Small Hart Chart suitable for near viewing
3. Eye patch

Setup

1. Occlude the participant’s left eye.
2. Place the large letter chart at 3 m.
3. Have the participant hold the small letter chart at 40 cm.

Procedure

1. Ask the participant to hold the small chart at 40 cm and call off the letters on the top line as he or she slowly moves the chart closer.
2. When he or she can no longer keep it clear have him move the chart 2.5 cm further away and then shift to the second line of the larger chart placed at about 3 m. Repeat the far to near change for each letter on the second line.
3. After completing line two, move the small chart to 33 cm and call off the letters on the third line as he or she slowly moves the chart closer. Then repeat step 2.
4. Repeat until all 10 lines are complete.
5. If the participant is experiencing difficulty at any level:
    a. Suggest that participant try and get the “feeling” of looking close and crossing his or her eyes for the small chart or have the participant try and get the feeling of relaxing or starring for the large chart.
6. Repeat the procedure with the patch moved to the right eye.

Endpoint

1. Successfully clear the near chart held at a distance of 33 cm and be able to clear the distant chart.
2. The participant should be able to appreciate the different feeling and effort associated with clearing the print while viewing the far chart compared with clearing the print while viewing the near chart.

2. Phase 2 Office Therapy Procedures List

Vergence Non-Computer
Vectograms (Spirangle)
Aperture Rule (base-out/base-in)

Vergence Computer-based
VTS4 (RDS) Base-out and base-in

Accommodation Techniques
Loose Lens Accommodative Rock (Level 2)
Letter Chart Accommodative Rock (Level 2)

2.1 Vectograms (Spirangle)

Objective

1. To increase positive fusional amplitudes.
2. To increase negative fusional amplitudes.

Equipment Needed

1. Spirangle Vectogram
2. Dual Polachrome Illuminated Trainer
3. Polaroid Glasses

Setup

The participant wears Polaroid glasses and the Spirangle Vectogram targets are set up in the Dual Polachrome Illuminated Trainer on the top level at 2 base-out and the Spirangle on the bottom level set at zero prismatic demand.

Procedure

Therapy

Explain to the participant that you are going to demonstrate the procedure that he or she will practice. It involves 2 distinct steps.

1. **Step One:** Tell the participant to separate the Spirangle sheets to 3Δ Base-out and maintain clear, single vision.
2. **Step two**: Have the participant start by looking at the letter "E" (easiest) in the middle and once fused to slowly move the eyes clockwise around the Spirangle, fusing each letter in order until reaching the letter "I" (hardest) and achieve clear, single vision
2. The participant should be able to appreciate the significant spiraling out effect of the Vectogram and point to each letter (localization) as they move from one letter to another.
4. The therapist should be able to notice that the participant is localizing closer and closer as the move around the Spirangle.
5. Then increase the disparity by 3Δ Base-out and continue until good performance at 25 Δ Base-out.
5. Repeat with base-in
6. If the participant is experiencing difficulty:
   a. Suggest that the participant get the feeling of looking close and crossing his or her eyes.
   b. Use the feedback technique of localization to show him how to regain fusion.

Endpoint

1. 25^{Δ} Base-out.
2. 12^{Δ} Base-in (Letter L).

2.2 Quoits with Head Movement

Objective

1. To increase positive and negative fusional amplitudes while the participant is moving the head..

Equipment Needed

1. Quoit Vectogram
2. Hand Held Horizontal Holder
3. Polaroid Glasses

Setup

The participant wears Polaroid glasses and the Quoit Vectogram targets are set up in the hand-held Vectogram holder at 10 base-out

Procedure

Therapy

Explain to the participant that you are going to demonstrate the procedure that he or she will practice. It involves 2 distinct steps.

1. **Step One:** Tell the participant to separate the Quoit sheets to 10Δ Base-out and maintain clear, single vision.
2. **Step two**: Have the participant continue to fuse the Quit while moving the head left and right and then, up and down.

Endpoint

1. no discomfort

2.3 *Aperture Rule*

Objective

To increase positive and negative fusional amplitudes

Equipment Needed

1. Bernell Aperture Rule Trainer and Cards
2. Pointer

Setup

1. Place the single aperture at the number one mark on the Aperture Rule and the cards at the setting marked "place aperture cards here".
2. The participant should place the end of the Aperture Rule against the bridge of his or her nose.

Procedure

Convergence Therapy

1. Turn to card number one.
2. Alternately cover the participant's right and left eyes and demonstrate that he or she will see one target with the right eye and one with the left eye.
3. Remove the cover paddle and ask the participant what he or she sees with both eyes open.
4. He or she will report double vision, suppression or will be able to fuse the two targets and report that he or she sees one target.
5. Explain that the objective is to achieve clear, single vision.
6. If he or she is unable to fuse you can use the same techniques that were suggested for the previous binocular vision therapy procedures. These include kinesthetic awareness of looking close or localization suggested in the Quoits Vectogram procedure.
7. To use the localization technique with the Aperture Rule have the participant hold the pointer directly behind the single aperture. Instruct him to look directly at the pointer. If he or she does this, he or she will report one pointer and one target. Once the participant realizes where he or she must look, try removing the pointer. With some practice the participant will soon be able to fuse without the additional support of the pointer.
8. Once fusion is reported, question the participant regarding the clarity of the target, the suppression cues, and whether he or she appreciates the depth in the circles.
9. Ask the participant to hold fusion for a count of five, look away momentarily and then try and regain fusion as quickly as possible.
10. This procedure should be repeated five times and the next card is then exposed, the single aperture is moved to its appropriate position and the entire procedure (steps 3-9) is repeated. Goal is card #12.

Divergence Therapy

The same general procedures are performed for divergence therapy, except that the double aperture slide is substituted for the single aperture slide and when attempting localization, the pointer needs to be behind the card.

Endpoint

Successfully achieve clear, single binocular vision with a prismatic demand of 30^{Δ} Base-out (Card 12) and 15^{Δ} Base-in (card 6)

2.4 VTS4 Random Dot Stereogram (RDS)

Objective

To increase positive and negative fusional amplitudes.

Equipment Needed

VTS4/Computer Orthopter

Setup

1. See. 1.3.5. The participant wears liquid crystal glasses.
2. At the main Menu select **“Multiple Choice Vergence”**
3. On the left hand side under “DIRECTION select **“horizontal” and “base out”**
4. On the right hand side of the screen select **“RDS”**
5. The others settings that you will see on the bottom of the screen do not require any adjustment.
6. Finally select the stimulus option with the “**blue box and the letter A**”.

1. The procedure will begin and last for 5 minutes

Procedure: Convergence Therapy

1. Ask the participant if he or she is able to see the Letter “A” in the middle of the large square and another smaller square to be floating closer than the large square. This smaller square will either be above, below, to the right, or to the left of the letter “A”.
2. The participant should be able to move the game pad button in the direction he or she sees the floating square (right, left, up down).
3. The software program increases the prismatic demand of the task after two correct responses and decreases the prismatic demand after an incorrect response. The participant will receive auditory feedback for either a correct or an incorrect response

Procedure: Divergence Therapy

1. Select Base-in vergence and repeat the procedure described above for use for Base-out vergence.

Endpoint

1. 45^{Δ} Base-out/15^{Δ} Base-in with RDS targets

2.5 Loose Lens Accommodative Rock (Level 2)

Objective

1. Restore normal monocular accommodative amplitude.
2. Restore normal monocular accommodative facility.

Equipment Needed

1. Hopping Accommodative Facility VT cards
2. Loose lenses from +2.00 D to -6.00 D in 0.50 D increments
3. Eye patch

Setup

1. Occlude the participant's left eye.
2. Hopping Accommodative Facility VT card is held at 40 cm.
3. The participant is asked to hold two lenses, one in the right hand and one in his or her left hand.
4. Start with +0.50 D and –0.50 D.

Procedure

1. The participant is asked to clear the print as he or she alternately holds the plus and then the minus lens in front of his or her right eye.
2. Give the participant as much time as necessary for him to clear and read the print.
3. The goal is to achieve clear vision, 10 times, without regard to the time factor.
4. Increase the power of the lenses in 0.50 D increments up to the endpoints (+2.00/-6.00).
5. If the participant is experiencing difficulty at any level:
   a. Suggest that participant try and get the "feeling" of looking close and crossing his or her eyes for the minus lenses or have the participant try and get the feeling of relaxing or starring for the plus lenses.
   b. Decrease the demand by moving card away until the print is clear and then moving back to 40 cm for the minus lenses or move the card closer until the print clears and then move back to 40 cm for the plus lenses.
   c. Decrease the demand by decreasing the power of the lenses for either plus or minus.
6. Repeat the procedure with the right eye occluded.

1. Once the participant can achieve clarity through +2.00/-6.00 lenses, begin to work on speed. The goal is to achieve 10 cpm with +2.00/-6.00.

Endpoint

1. Clear +2.00/-6.00, 10 cycles in one minute.
2. The participant should be able to appreciate the different feeling and effort associated with clearing the print through minus and plus lenses.

2.6 Letter Chart Accommodative Rock (Level 2)

Objective

1. Restore normal monocular accommodative amplitude
2. Restore normal monocular accommodative facility

Equipment Needed

1. Large Hart Chart suitable for distance viewing
2. Small Hart Chart suitable for near viewing
3. Eye patch

Setup

1. Occlude the participant's left eye
2. Place the large letter chart at 3 m
3. Have the participant hold the small letter chart at 40 cm

Procedure

1. Ask the participant to hold the small chart at 40 cm and call off the letters on the top line as he or she slowly moves the chart closer.
2. When he/she can no longer keep it clear have the participant move the chart 2.5 cm further away and then shift to the second line of the larger chart placed at about 3 m. Repeat the far to near change for each letter on the second line.
3. After completing line two, move the small chart to 40 cm and call off letters on the third line as he/she slowly moves the chart closer. Then repeat step 2.
4. Repeat until all 10 lines are complete.
5. If the participant is experiencing difficulty at any level:
   a. Suggest that participant try and get the "feeling" of looking close and crossing his/her eyes for the small chart or have the participant try and get the feeling of relaxing or staring for the large chart.
6. Repeat the procedure with the patch moved to the right eye.

Endpoint

1. Successfully clear the near chart held at a distance equal to age-appropriate amplitude and is able to do 10 cycles per minute of far to near fixations. The distance for age-appropriate amplitude should be calculated using the formula 18.5 – 0.3 x age.
2. The participant should be able to appreciate the different feeling and effort associated with clearing the print while viewing the far chart compared with clearing the print while viewing the near chart.

3. Phase 3 Office Therapy Procedures List

Fusional Vergence
Vectograms (Quoits/Spirangle) Jump Vergence
Eccentric Circles
VTS4 (RDS) Step Jump Vergence

3.1 Vectograms (Quoits/Spirangle) Jump Vergence

Objective

1. To increase positive fusional amplitudes.
2. To increase negative fusional amplitudes.

Equipment Needed

1. Quoit Vectogram
2. Spirangle Vectogram
2. Dual Polachrome Illuminated Trainer
3. Polaroid Glasses

Setup

The participant wears Polaroid glasses and the Quoit Vectogram targets are set up in the Dual Polachrome Illuminated Trainer on the top level at 2 base-out and the Spirangle on the bottom level set at zero prismatic demand.

Procedure

Therapy

Explain to the participant that you are going to demonstrate the procedure that he or she will practice. It involves 2 distinct steps.

1. **Step One:** Tell the participant to separate the Quoit sheets to 2Δ Base-out and maintain clear, single vision.
2. **Step two**: Have the participant look down to the Spirangle and achieve clear, single vision
2. Once the participant can perform these steps easily for 3 cycles, increase the BO demand by 2 and the BI demand by 1 and repeat, looking up and down from the top Vectograms to the bottom Vectograms.
4. If the participant is experiencing difficulty:
   a. Suggest that the participant get the feeling of looking close and crossing his or her eyes.
   b. Use the feedback technique of localization to show him how to regain fusion.

1. all.
2. If he cannot, have him walk 6 inches closer or farther away until he does experience fusion.
3. Continue separating the targets by 3 letters at a time until the participant is able to fusion the Quoits at letter “L”.

Endpoint

1. 25^{Δ} Base-out.
2. 12^{Δ} Base-in (Letter L).

3.2 Eccentric Circles

Objective

1. To increase positive fusional amplitudes
2. To increase negative fusional amplitudes

Equipment Needed

1. Keystone Opaque Eccentric Circles
2. Keystone Transparent Eccentric Circles
3. Pointer

Setup

1. Either have the participant hold the cards or place them in the Polachrome Illuminated Trainer, the horizontal holder or any other suitable device.
2. The cards should be held about 40 cm from the participant.
3. Begin with the two cards together with the "A"s touching.

Procedure

Convergence Therapy

1. The participant should see two cards at this point.
2. Ask the participant to try and cross his/her eyes and get the feeling of looking closer.
3. If he/she cannot do this voluntarily, use localization with a pointer to demonstrate the point to which he/she must converge to achieve fusion.
4. Tell the participant that when he/she achieves fusion he/she will see "three sets of circles."
5. Explain that he/she is to concentrate only on the middle set and is to ignore the two side images.
6. Ask him/her about the middle set of cards. He/she should be able to spontaneously indicate that he/she sees two circles, one larger than the other and that the larger one appears to be floating closer to him/her. In addition, he/she should see the word "clear", in focus. If he/she does not spontaneously respond with this information, ask the participant to relax their focus a little or maintain fusion for 5 seconds and see if the letters clear. It is important to make the participant aware that this perception of depth is a feedback cue about his/her performance.
7. Once he/she can achieve fusion ask him/her to hold the position for 5 seconds, look away momentarily, and look back at the cards and regain fusion. Instruct the participant to repeat these 10 times and then separate the cards about 1 centimeter and repeat the entire procedure again. Continue until he/she is able to achieve fusion, look away and back with the cards separated about 12 cm (measure from the left side of the outer ring on the left card to the left side of the outer ring on the right card).

Divergence Therapy

1. The same general procedures are performed for divergence therapy except that the participant must now diverge behind the plane of the cards.
2. Because it is difficult for some participants to visualize looking behind an opaque object, translucent Eccentric Circle cards are available for divergence therapy.
3. Participants often experience some difficulty initially with this procedure. It is helpful to show them where they must look to achieve orthopic fusion. To accomplish this tape a pointer to the

wall at the participant's eye level. Have the participant stand about 3-4 feet away from the wall and hold the transparent Eccentric Circle Cards about 25 cm away from his/her eyes.

4. Instruct the participant to look at the pointer on the wall and while doing so to be aware of the circles. He/she should be able to see three sets of circles. If he/she cannot, have the participant walk closer or farther away until he/she does appreciate three circles. Tell the participant to concentrate on the middle set and ignore the side images. Once he/she can achieve this have the participant repeat the same procedures described for convergence therapy.
5. The only difference is that because of the lower physiological limit for divergence the final separation will be smaller. Fusion with a 6 cm separation is considered adequate (measure from the left side of the outer ring on the left card to the left side of the outer ring on the right card).

Endpoint

At a working distance of 40 cm, achieve clear, chiastopic fusion with a card separation of 12 cm (30^{Δ} Base-out) and clear, orthopic fusion with a card separation of 6 cm (15^{Δ} Base- in). Measure separation from the left side of the outer ring on the left card to the left side of the outer ring on the right card.

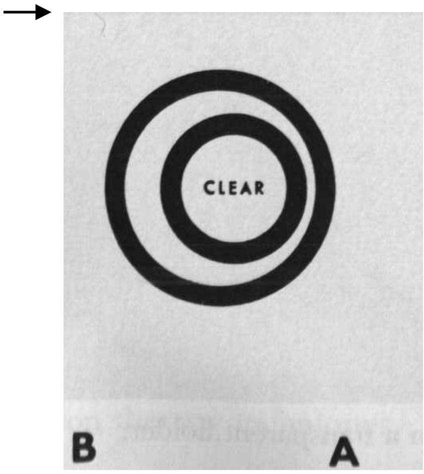

12cm separation for base-out

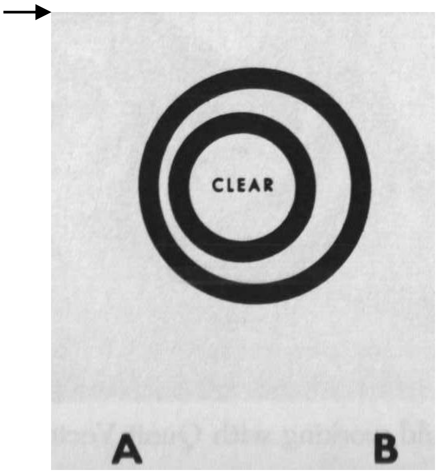

3.3 VTS4/Step Jump DuctionsRDS (Phase 3)

Objective

1. To increase the speed and decrease the latency of the positive fusional vergence response.
2. To increase the speed and decrease the latency of the negative fusional vergence response.

Objective

To increase positive and negative fusional amplitudes.

Equipment Needed

VTS4

Setup

1. The participant wears liquid crystal glasses.
2. At the main Menu select **"JUMP DUCTION"**
3. On the left hand side under "DIRECTION select **"horizontal" and "BO + BI"**
4. Select **"STEP"**

4. On the right hand side of the screen select **"RDS"**
5. The others settings that you will see on the bottom of the screen do not require any adjustment.
6. Finally select the stimulus option with the "**blue box and the letter A**".

1. The procedure will begin and last for 5 minutes

Procedure:

1. Ask the participant if he or she is able to see the Letter "A" in the middle of the large square and another smaller square to be floating closer than the large square. This smaller square will either be above, below, to the right, or to the left of the letter "A".
2. The participant should be able to move the game pad button in the direction he or she sees the floating square (right, left, up down).
3. The software program changes the demand from base-in to base-out after each response and increases the prismatic demand of the task after two correct responses and decreases the prismatic demand after an incorrect response. The participant will receive auditory feedback for either a correct or an incorrect response.
4. When participant can fuse 45^{Δ} Base-out to 15^{Δ} Base-in for at least 15 repetitions in one minute repeat above with red box with the letter "A" selected (small targets).

Endpoint

Participant can fuse 45^{Δ} Base-out to 15^{Δ} Base-in for at least 15 repetitions in one minute using small targets in step jump duction mode.

4. Phase 4 Office Therapy Procedures List

Fusional Vergence
VTS4 (RDS) Jump Vergence
Eccentric Circles Jump Vergence

Binocular Accommodation
Aperture Rule/Flippers

4.1 VTS4 (RDS) Jump Vergence (Phase 4)

***VTS4/ Jump-Jump Ductions* (RDS) (Phase 4)**

Objective

1. To increase the speed and decrease the latency of the positive fusional vergence response.
2. To increase the speed and decrease the latency of the negative fusional vergence response.

Objective

To increase positive and negative fusional amplitudes.

Equipment Needed

VTS4

Setup

1. See 1.3.5. The participant wears liquid crystal glasses.
2. At the main Menu select **"JUMP DUCTION"**
3. On the left hand side under "DIRECTION select **"horizontal" and "BO + BI"**
4. Select **"RANDOM"**

4. On the right hand side of the screen select **"RDS"**
5. The others settings that you will see on the bottom of the screen do not require any adjustment.
6. Finally select the stimulus option with the "**blue box and the letter A**".

1. The procedure will begin and last for 5 minutes

Procedure:

1. Ask the participant if he or she is able to see the Letter "A" in the middle of the large square and another smaller square to be floating closer than the large square. This smaller square will either be above, below, to the right, or to the left of the letter "A".
2. The participant should be able to move the game pad button in the direction he or she sees the floating square (right, left, up down).
3. The software program changes the demand from base-in to base-out after each response and increases the prismatic demand of the task after two correct responses and decreases the prismatic demand after an incorrect response. The participant will receive auditory feedback for either a correct or an incorrect response
4. When participant can fuse 45^{Δ} Base-out to 15^{Δ} Base-in for at least 15 repetitions in one minute repeat above with red box with the letter "A" selected (small targets).

Endpoint

Participant can fuse 45^{Δ} Base-out to 15^{Δ} Base-in for at least 15 repetitions in one minute using small targets in Random jump duction mode.

4.2 Eccentric Circles Jump Vergence

Objective

To increase the speed and decrease the latency of the positive and negative fusional response.

Equipment Needed

1. Keystone Opaque Eccentric Circle Cards
2. Keystone Transparent Eccentric Circle Cards
3. Pointer

Setup

1. Either have the participant hold the cards or place them in the Polachrome Illuminated Trainer, the horizontal holder or any other suitable device.
2. The cards should be held about 40 cm from the participant.
3. Begin with the two cards together with the "A"s touching.

Jump vergence Procedures

1. Ask the participant to fuse the Eccentric Circle Cards using chiastopic fusion, then look away for several seconds and look back and regain fusion.
2. After the participant has fused the Eccentric Circle Cards, then cover one eye for 5 seconds to break fusion. He or she then uncovers his or her eye and has to regain fusion.

Jump Divergence Procedures

1. Ask the participant to fuse the Eccentric Circle Cards using orthopic fusion, then look away for several seconds and look back and regain fusion.
2. After the participant has fused the Eccentric Circle Cards, then cover one eye for 5 seconds to break fusion. He or she then uncovers his or her eye and has to regain fusion.

Convergence to Divergence Procedure

1. With the cards held together, ask the participant to switch from chiastopic to orthoptic fusion. He or she should hold fusion for about 5 seconds chiastopically and then switch to orthoptic fusion and hold for 5 seconds.
2. This should be repeated 20 times
3. Separate the cards 1 cm and repeat steps 1-3
4. Continue until a separation of 6 cm can be achieved

Endpoint

1. Regain clear, chiastopic fusion after fusion is disrupted with a card separation of 12 cm (30^{Δ} Base-out) and clear, orthopic fusion with a card separation of 6 cm (15^{Δ} Base-in).
2. Switch between chiastopic and orthopic fusion with the cards held 6 cm apart for 20 repetitions

4.3 Aperture Rule/Flippers

Objective

To decrease the latency and increase the speed of the accommodative response under binocular conditions

Equipment Needed

1. Lens flippers in various powers: +/-1.00, +/-1.50, +/-2.00.
2. Aperture Rule with single aperture

Setup

1. The Aperture Rule is set at card 2 with the single aperture in place
2. The participant holds the +1/-1 flipper in front of his/her eyes.

Procedures

1. Lens flippers (+1.00/-1.00) are held before the participant's eyes with the "minus" lenses as a starting point.
2. Explain that the objective is to achieve clear, single vision.
3. If he or she is unable to fuse you can use the same techniques that were suggested for the previous binocular vision therapy procedures. These include kinesthetic awareness of looking close or localization suggested in the Quoits Vectogram procedure.
4. Once fusion is reported, question the participant regarding the clarity of the target, the suppression cues, and whether he or she appreciates the depth in the circles.
5. Ask the participant to hold fusion for a count of five, and then flip the flipper to the other side (plus lens) and then try and regain fusion as quickly as possible.
6. This procedure should be repeated five times and the next card is then exposed, the single aperture is moved to its appropriate position and the entire procedure (steps 3-9) is repeated. Goal is card #12.

Endpoint

Able to achieve single, clear, vision while viewing Aperture Rule Card 6 through +2.00 and -2.00 lenses for at least 13 cycles per minute without suppression

5. Home Therapy Equipment and Sequence

Equipment

Brock String
Keystone Transparent Eccentric Circles
Opaque Life Saver Cards
HTS disk

Therapy Sequence

Sessions 1 to 3

Procedure	Minutes assigned
Brock String (Level 1)	5
HTS	10

Sessions 4 to 6

(5-8 session)

Procedure	Minutes assigned
Brock String (Level 2)	5
HTS	10

Sessions 7 to 9

Procedure	Minutes assigned
Brock String (Level3)	5
HTS	10

Sessions 10 to 12

Procedure	Minutes assigned
Eccentric Circle	5
HTS	10

Sessions 13 to 16

Procedure	Minutes assigned
Eccentric Circle Jump Vergence	5
HTS	10

Brock String (Level 1) – Home Instructions

Objective

To develop your awareness of crossing your eyes.

Equipment Needed

1. Brock String

Procedure

1. Use two beads and about 1m of string.
2. Attach one end of the string on a chair or doorknob and hold the other end of the string taut and against the bridge of your nose.
3. Set the red bead at 2 ft (60 cm) from your nose and the green bead at about 1 ft (30 cm).
4. Look at the closer bead (green bead) and observe what you see and appreciate how your eyes feel. You should see one green bead and two red beads. In addition, you should notice two strings crossing at the green bead (looking like the letter X) with one string coming from your right eye and the other coming from your left eye. You should appreciate that your eyes are crossing or working harder.
5. Now look at the far bead (red bead) and you should now see one red bead with the strings crossing (looking like the letter X) at the red bead. You will also notice two green beads. You should appreciate that now your eyes are more relaxed than when looking at the green bead.
6. Remember the strings should look as if they are extensions of your right and left eyes. Where you perceive the two strings crossing is actually where your eyes are aimed. Thus, if you are trying to look at the green bead but the strings appear to cross farther away than the bead, this is an indication that you are looking too far away. Use this information to try and correct your eye position and look closer.
7. If you have trouble:
   a. Try and get the "feeling" of looking close, crossing your eyes, or working harder.
   b. Touch the bead that you are trying to make single..
8. Once you are able to fuse the near and far beads, try to keep the near bead single for 5 seconds and then switch to the far bead and hold for 5 seconds. Repeat this 10 times
9. Then move the near bead 2 inches (5 cm) closer while always maintaining the far bead at 2 feet (60 cm) and repeat step 5.
10. Continue moving the near bead closer until you reach your therapy goal.

Your Therapy Goal!

1. Move the near bead closer until you can successfully cross your eyes at a distance of 2 inches (5 cm) from your nose. You should be able to quickly move back and forth from the near bead to the far bead.
2. You should also be able to appreciate the different "feeling" and effort associated with crossing and relaxing your eyes.

Brock String (Level 2) – Home Instructions

Objective

To improve the control of your ability to cross and relax your eyes

Equipment Needed

1. Brock String

Procedure

1. Use about 1 m of string and place one bead at 2.5 cm and the other at 1 m.
2. Attach one end of the string on a chair or doorknob and hold the other end of the string taut and against the bridge of your nose.
3. Look at the bead at 1 m away and try and see that the two strings meet at that bead.
4. Now very slowly look closer and closer (as if a bug is walking on the string towards you) until you are looking at the bead 2.5 cm in front of your nose. Repeat this 10 times.
5. If you have trouble:
   a. Try and get the "feeling" of looking close, crossing your eyes, or working harder.
   b. Touch the point on the string that you are trying to look at with your finger or pencil tip and move it along the string towards your nose. As it gets easier remove your finger or the pencil.
6. After you can converge all the way to within 5 cm of your nose, slowly look along the string from that point to the end of the string. Repeat going towards and away from your nose 20 times.
1. Finally remove all beads from the string and slowly cross and relax your eyes, making sure you see the "X" at all times.

Your Therapy Goal!

1. You should be able to cross your eyes to within 2.5 cm of your nose and relax your eye to 1 m.
2. You should be able to slowly look along the string from near your nose to the end of the string and back again.
3. You should also be able to appreciate the different "feeling" and effort associated with crossing and relaxing your eyes.

Brock String (Level 3) – Home Instructions

Objective

Develop the ability of your eyes to voluntarily converge

Equipment Needed

3. None

Setup

No setup required

Procedure

1. You should be able to voluntarily cross your eyes without the aid of a pointer, finger or any other object. To do so you should try and imagine an object slowly moving towards your eyes and try to get the feeling of crossing your eyes like when you worked with the Brock string.
4. Once you can cross your eyes so that you are looking as close as 1-2 inches from your nose, reverse the process and gradually relax your eyes and look at a distance target. Repeat this procedure for 20 repetitions.

Endpoint

1. You should be able to voluntarily cross your eyes to a distance 2.5 cm (I inch) from your nose.
2. You should be able to appreciate the different feeling and effort associated with crossing and relaxing your eyes.

Home Therapy System (HTS) – Home Instructions

Objective

To improve your ability to comfortably cross and relax your eyes.

Equipment Needed

1. Computer
2. HTS program disk
3. Red and Blue glasses

Procedure

1. To launch the HTS program double click on the HTS icon that has been added to your Desktop screen.
2. The first time you use the HTS program you will be prompted to enter your name. Once your name has been entered it cannot be changed.
3. Follow the prompts on the screen that lead you step by step though a number f preliminary setup procedures (measure screen size, indicate whether you have internet access)
4. Once the setup is complete you can begin the home therapy.
5. Place red and blue glasses over your eyes and click on the "**Run Program"** button and chose "Classic option"
6. You should notice a large red square with a smaller square inside that is "popping out" towards you.
7. Move the arrow key to the side where the small square appears (i.e., push left arrow if the small square is on the left side of the larger square).
8. If you correctly match the location of the smaller square the computer will "beep" at you and increase the difficulty. If you are incorrect the computer will "boop" and decrease the difficulty.
9. When you reach the point where the large box separates, do not arbitrarily push the arrow keys.
10. Try to get the "feeling" of pulling your eyes together, crossing your eyes, or working harder until the boxes fuse together.
11. Repeat the procedure for the allotted practice time.
12. As you complete each one of the assigned procedures, the computer program will automatically move to the next procedure. For example, after completing the Vergence Base-out for the allotted time, Vergence Base-in will begin. For Base-in Vergence, try to get the "feeling" of relaxing your eyes together, staring in the distance, looking far away.
13. You can also select tow other options (clicker or space ship for variety).

Your Therapy Goal!

Your therapy goal is to complete each assigned activity in the HTS program.

Eccentric Circles - – Home Instructions

Objective

To improve your ability to cross your eyes quickly and efficiently.

Equipment Needed

1. Transparent Eccentric Circle Cards
2. Pointer

Convergence Procedures

1. Hold the cards about 16 inches (40 cm) from your nose.
2. Begin with the two cards together with the "A"s touching.
3. Start by getting the three circles as you learned in the office.
4. Once you are able to hold the three circles for 5 seconds, look away and look back, trying to get the three circles back as soon as possible.
5. If you are having trouble try the following:
    a. Place a pencil tip slightly below and between the two cards.
    b. Stare at the pencil tip.
    c. Slowly move the pencil tip away from the cards and closer to you while looking at the pencil tip the whole time.
    d. As you pull the pencil tip closer, you should NOTICE that the two cards on either side of the pencil are getting blurry and may start to split apart. Remember to keep your eyes on the pencil tip as you pull it closer to you.
    e. Keep moving the pencil tip toward you until you notice the inside pair of cards merge and create a third card with two circles directly under your pencil tip.
    f. Try to clear that third card by slowly moving the pencil slightly forwards and backwards until you get it clear. Ignore all the other two cards on the sides.
    g. The outer circle will appear to float closer to you than the inner circle.
    h. Once you are able to hold the three circles for 5 seconds, look away and look back, trying to get the three circles back as soon as possible.
    i. Take the pencil away and try to keep that card clear and single.
    j. Hold for another ten seconds.
    k. You may relax your eyes now.
    l. Try this again without the use of the pencil. If you have difficulties continue to use the pencil.
6. Once you can do this with the cards together begin to increase the separation ¼ inch at a time and repeat the process.
7. Continue until you can do the above with a 5 inch separation of the cards.

Your Therapy Goal!

You should be able to see three cards, the word “CLEAR” in the middle card should be clear, the outer circle in the middle card should float closer to you than the inner circle. You should be able to do this with the cards separated by 5 inches.

Divergence Procedures

1. Hold the CLEAR Eccentric Circle cards about 16 inches (40 cm) from your nose.
2. Begin with the two cards together with the "A"s touching.

3. Start by getting the three circles as you learned in the office. Remember that for divergence you must relax your eyes and look through the clear cards at a distance.
4. Once you are able to hold the three circles for 5 seconds, look away and look back, trying to get the three circles back as soon as possible.
5. If you are having trouble try the following:
    a. Try to stare out at a distance object.
    b. It is helpful to tape a pencil or pick-up stick on the wall and sit about 10 feet away. Look through the Eccentric Circle cards at the stick on the wall.
    c. Once you are able to hold the three circles for 5 seconds, look away and look back, trying to get the three circles back as soon as possible.
6. Once you can do this with the cards together begin to increase the separation ¼ inch at a time and repeat the process.
7. Continue until you can do the above with a 2.5 inch separation of the cards.

Your Therapy Goal!

You should be able to see three cards, the word "CLEAR" in the middle card should be clear, the inner circle in the middle card should float closer to you than the outer circle. You should be able to do this with the cards separated by 2.5 inches.

Eccentric Circle Jump Vergence - – Home Instructions

Objective

To improve your ability to cross and relax your eyes quickly and efficiently.

Equipment Needed

1. Transparent Eccentric Circle Cards
2. Pointer

Procedures

1. Hold the cards about 16 inches (40 cm) from your nose.
2. Begin with the two cards together with the "A"s touching.
3. Start by using convergence hold the three circles for 5 seconds, the switch to divergence to get the three circles back as soon as possible.
4. Notice the difference in which circle is coming closer to you when you switch from convergence to divergence.
5. Continue switching between convergence and divergence until it takes very little effort to get the third circle.

Your Therapy Goal!

You should be able to, getting the third circle as quickly as possible, switch between convergence and divergence 20 times in 1 minute.

Appendix

Office Based Vergence/Accommodative Therapy (OBVAM)

- **Select first procedure from each category and continue with these procedures until the endpoint is achieved. After achieving endpoint select next procedure in category.**
- **Date and initial each procedure as it is completed, and record date procedure is completed**

PHASE ONE (6 Visits)		
**Concussion-Related Saccadic Therapy**		**Date & Initial**
Level 1: 2 Pencils (still) – Horizontal (head still), slow to fast repeat Vertical repeat for Vergence (far and near)	no discomfort (1 min each eye movement type) 3 min total plus instruction	
Level 2: 2 Pencils (moving) – Horizontal (head still), slow to fast repeat Vertical and repeat Vergence (far and near)	no discomfort (1 min each eye movement type) 3 min total plus instruction	
Level 3: 2 Pencils (still & repeat moving) – Horizontal (head still), slow to fast walking forward and backward for saccade Horizontal/ Vertical and repeat Vergence	no discomfort	
Level 4: 2 Pencils –slow to fast walk in circle repeat activities of level 3	no discomfort	
Level 5: 2 Pencils –slow to fast rotating body (spinning top) repeat activities of level 3	Minimal discomfort (slight dizziness is ok)	
**Concussion-Related Pursuit Therapy**		
Level 1: Hanging Ball –left to right, body still	no discomfort	
Level 2:Hanging Ball –forward and backward, body still	no discomfort	
Level 3: Hanging Ball - body rocking L to R, opposite direction of ball movement<br>Repeat for vergence F and B, opposite direction of ball	no discomfort	
Level 4: Hanging Ball – Circle around swinging ball	no discomfort	
**Gross Convergence**	**Endpoint**	**Date & Initial**
Brock String (Level 1)	Convergence to a bead placed 2.5 cm from nose	
Brock String (Level 2)	Voluntarily converge to 2.5 cm from nose	
Brock String Level 3	Converge eyes with no string	
**Accommodation**	**Endpoint**	**Date & Initial**
Loose Lens Accommodation Rock (Lev	Clear +1.50/-3.00, 10 cycles per minute	

1)		
Letter Chart Accomm Rock (Level 1)	Clear near chart at 33 cm, change fixation and clear far Letter Chart at 3m for 10 cycles per minute	
**Vergence Non-Computer**	**Endpoint**	**Date & Initial**
Vectograms (Quoits, Base-out/Base-in (3/1 ratio)	25 Δ Base-out/12 base-in (letter L)<br>Every cm is 2.5prismdiopter	
**Vergence -Computer-Based**	**Endpoint**	**Date & Initial**
VTS4 (RDS) Base-out only	30 base-out with RDS large targets (blue)	
**PHASE TWO (4 Visits)**		
**Accommodation**	**Endpoint**	**Date**
Loose Lens Accomm Rock (Level 2)	Clear +2.00/-6.00, 10 cycles per minute<br>MAX +2.5 to whatever they can handle<br>-10D	
2 Letter Chart Accomm Rock (Level 2)	Clear near chart at age-appropriate distance and be able to clear to distance chart (difficulty is where the near card is placed and be about 10 cm from the eye ball)	
**Vergence Non-Computer**	**Endpoint**	**Date**
Vectograms (Spirangle) Base-out/Base-in (3/1 ratio)	25 Δ Base-out/12 base-in (letter L)	
Aperture Rule	30 Δ Base-Out (Card 12), 15Δ Base-in (Card 6)	
Eccentric Circles	30 Δ Base-out (12 cm)	
**Vergence -Computer-Based**	**Endpoint**	**Date & Initial**
VTS4 RDS Base-out/Base-in (3/1 ratio)	45Δ Based out with RDS large targets / 15 Base-in, repeat with small targets	
**PHASE THREE (3 Visits)**		
**Vergence Non-Computer**	**Endpoint**	**Date & Initial**
Vectograms (Quoits/Spirangle) Jump Vergence	25 Δ Base-out, 12Δ Baseline (Letter “L”)<br>(alternately fuse from top to bottom Vectogram	
Vectograms (Quoit Base-out) with head movement (left, right, up, down) with patient sitting (no more than 2 inch movement each way)	No discomfort, up to 10 base-out	
Eccentric Circles	30 Δ Base-out (12 cm), 15 Δ (6 cm) Base-in	
**Vergence -Computer-Based**	**Endpoint**	**Date & Initial**
VTS4 (RDS) Jump Vergence	Fuse 45Δ Base out to 12Δ Base-in for at least 15 repetitions in 1 minute in *Step jump* duction mode with small targets	
**PHASE Four (3 Visits)**		

Accommodation	Endpoint	Date & Initial
Aperture Rule / Flippers	Single, clear vision while viewing Card 6 through +2.00 and alternately -2.00 at least 13 cycles per minute without suppression	
**Vergence Non-Computer**	**Endpoint**	**Date & Initial**
Eccentric Circles Jump Vergence	• Jump from BO fusion (separation of 12 cm, 30Δ Base-out) to BI fusion (separation of 6cm, 15Δ Base-in).<br>• Switch between BO/BI fusion with the cards held 6 cm apart for 20 repetitions	
Vectograms walking forward and backward at 10 BO	No discomfort, up to 10 base-out (5 steps, 3 cycles F&B)	
Vectograms walking in a circle at 10 BO	No discomfort, up to 10 base-out	
**Vergence -Computer-Based**	**Endpoint**	**Date & Initial**
VTS4 (RDS) Jump Vergence	Fuse 45 Δ Base-out to 15Δ Base-in for at least 15 repetitions in 1 minutes in *random jump* duction mode	

By dating a specific procedure, you are certifying that the participant has achieved the desired endpoint for that procedure.
