# Supplementary material for: Effectiveness of treatment for concussion-related convergence insufficiency: The CONCUSS study protocol for a randomized clinical trial
Source: PLoS One. 2024 Nov 15;19(11):e0314027. doi: 10.1371/journal.pone.0314027 (PMC11567536; doi:10.1371/journal.pone.0314027)
Supplement: S1 File — (PDF) [file pone.0314027.s003.pdf]

Informed Consent

AAA

Please complete the survey below.

Thank you!

Informed Consent and HIPAA Authorization Form

Study Title: **Functional Mechanism of Neural Control in Post-Concussion Convergence Insufficiency**

Version Date: **October 28, 2022**

Principal Investigator: **Tara Alvarez, Ph.D.** **Telephone: (973) 596-5272**

You, or your child, may be eligible to take part in a research study. This form gives you important information about the study. It describes the purpose of this research study, and the risks and possible benefits of participating.

In the sections that follow, the word "we" means the study doctor and other research staff. If you are a parent or legal guardian who is giving permission for a child, please note that the word "you" refers to your child.

Study Overview

You or your child are being asked to take part in this research study because you have a concussion with lingering symptoms such as eye strain, double vision, headaches, difficulty reading, and blurred vision and are between the ages of 11-35 years old.

The purpose of this study is to find out if a type of vision therapy called "office-based vergence/accommodative therapy (OBVAT)" works better than the usual concussion care for helping to improve visual concussion symptoms.

This specific type of vision therapy has been shown to improve visual symptoms in people who's symptoms are not concussion related.

If you agree to take part, your participation will last for up to 17 weeks (about 4 months) and will involve 20-24 study visits (4-8 research visits and 16 OBVAT sessions). When you are asked to do OBVAT, you will see a vision therapist for one-hour sessions twice a week. There are differences between this study and your usual care. As a participant in the research you will:

- Complete 16 sessions of OBVAT and some at home exercises
- Complete 3 vision examinations
- Have 3-5 extra research visits
- Have 3-4 research MRI sessions
- Complete vision tasks
- Complete Cardiovascular tasks
- Complete symptom questionnaires

The main risks of this study are no more than minimal and limited to possible temporary increased or worsening of concussion symptoms from the visual therapy and exams.

You may benefit if OBVAT proves to be effective in improving your concussion symptoms.

If there is anything in this form you do not understand, please ask questions. Please take your time. You do not have to take part in this study if you do not want to. If you take part, you can leave the study at any time. If you do not choose to take part in this study, you can discuss treatment options with your doctor.

Please see below for additional details about the study.

What is involved in the study?

IRB#: 2210026053 APPROVED: 20-Oct-2023 EXP: 19-Oct-2024

If you agree to take part, you will complete 16 one-hour OBVAT sessions, either immediately or delayed (after 6 weeks of your usual concussion care recommended by your doctor, such as rest from screens and other similar activities). You will be randomly assigned to either the immediate or delayed group. During your time in this study, you will continue to see your doctor treating you for your concussion and follow their treatment plan. During your OBVAT therapy, you will see a vision therapist for a one hour session twice a week, and they will lead you through a series of vision exercises. If you are assigned to the immediate OBVAT group, you will complete a set of assessments 3 times: first prior to beginning the OBVAT, a second time after 12 sessions of OBVAT, and a third time after 4 final sessions of OBVAT. If you are assigned to the delayed OBVAT group, you will complete the same set of assessments at 3 different time points: first at a visit scheduled shortly after completing this consent visit, a second time after 6 weeks of your usual concussion care, and a third time after 16 OBVAT sessions (you will begin these OBVAT sessions after completing the set of assessments the second time). Both groups have the same amount of vision therapy.

What are the study procedures?

Some of the procedures in this study will be repeated several times. Tests that are part of your regular, routine medical care will continue to be performed. The study involves the following tests and procedures.

Randomization and Intervention: Your assignment for immediate OBVAT or delayed OBVAT will be determined at random, similar to a 'coin toss' as you will have an equal chance of being assigned into either group. Based on your group, you will either immediately begin OBVAT or complete 6 weeks of SCC prior to beginning OBVAT. For your OBVAT sessions, you will see a vision therapist for one hour twice a week. During these OBVAT sessions, you will perform eye exercises that are designed to train the connection between the eyes and the brain and help the eyes focus. Some examples of exercises that may be used are: focusing on an object as it moves closer or further away from your face, keeping your eyes focused on an object while moving your head, and switching focus between two objects.

Eye Exam: An optometrist will perform a standard eye exam. During this exam, the optometrists will measure how well your eyes focus and work together as a team and test your depth perception and eye movements. The exam will take approximately 1 hour.

Vision Tasks: You will be asked to wear a set of goggles that will track your eye movement while you are asked to perform two sets of vision tasks, one lasting about 15 minutes and the other lasting about 20 minutes. These tasks will be similar to some of those you complete as part of the eye exam. You may be asked to focus on certain images, follow some images or objects with your eyes as they move, and perform specific eye movements such as looking back and forth, side to side, or up and down.

Cardiovascular: You will have your electrocardiogram, blood pressure and flow with carbon dioxide measurements taken during the vision tasks and then lying down.

MRI: You will also have an MRI scan of the head. The MRI scan will be obtained at the Rutgers University Brain Imaging Center. MRI is an imaging technique that takes a picture of your brain. During the MRI, you will complete additional vision tasks. Before the MRI imaging session, a practice pretend scan will be performed so you can see what the vision tasks during the MRI are like.

During the MRI exam you will lie down on a bed (the MRI scanner) inside a tunnel that is open at each end. You will be asked to perform some vision tasks during this MRI similar to the vision tasks explained above. During the MRI exam you will hear loud banging noises. You are required to wear ear plugs to protect your ears.

MRI scans will be performed on routine clinical MRI scanners in the Department of Radiology. All of the MRI sequences used in this study are FDA-approved. Contrast and sedation will not be used.

If you are randomized to group 1, you will undergo 2 separate MRIs during the study. If you are randomized to group 2, you will undergo 3 separate MRIs during the study.

Pregnancy Testing: : If you are female, you will be offered and encouraged to take a urine pregnancy test prior to each MRI. You may refuse the pregnancy test if you so choose. If you choose not to take the pregnancy test, you will be asked to sign a Pregnancy Test Release Form stating that you have been informed of the potential risks of an MRI to a fetus and have been offered and refused a pregnancy test.

Questionnaires: You will be asked to fill out questionnaires to tell us how you are feeling as you recover from your concussion.

IRB#: 2210026053 APPROVED: 20-Oct-2023 EXP: 19-Oct-2024

At-home Reinforcement: While you are completing your OBVAT, you will also be asked to complete at-home reinforcement 3 days per week on days you are not doing OBVAT. You will be given a code with the reinforcement software on it. Each at-home reinforcement session will last 10-15 minutes. The software will log the number of sessions and minutes you complete.

Review of Medical Records: We will review your medical records. This will include prior history, recovery and follow-up information. Your records will be reviewed throughout the duration that you receive clinical care for this concussion.

Visit Schedule

The table below provides a brief description of the purpose and duration of each study visit for the immediate OBVAT group.

Visit	Purpose	Main Procedures	Duration
Visit 1*	Screening visit	Screening consent, interview, medical record review, vision assessment, informed consent, randomization, assignment to SCC+OBVAT or SCC alone.	30 minutes or until all questions about the study are answered
Visit 2-3*	Initial Assessment	Optometric clinical exam, vision tasks with cardiovascular, questionnaires	Either one 90 minute visit with a break OR<br>One 60 minute (visit 2) and one 30 minute visit (visit 3)
Visit 3 or 4	Initial imaging assessment	MRI	60 minutes
Visits 4-15	Therapy visits	60 minute visit with vision therapy procedures	60 minutes
Visit 16	Outcome visit 1	Optometric clinical exam, vision tasks with cardiovascular, questionnaires	60-75 minutes with break
Visit 17	Outcome visit 1 Imaging assessment	MRI session	60 minutes
Visits 17-20	Therapy visits	60 minute visit with vision therapy procedures	60 minutes
Visit 21	Outcome visit 2	Optometric clinical exam exam, vision tasks with cardiovascular, questionnaires	60-75 minutes with break
Visit 22	Outcome visit 3 (about one year later)	Optometric clinical exam, vision tasks with cardiovascular, questionnaires	60-75 minutes with break
Visit 23	Outcome visit 3 (about one year later)	MRI session	60 minutes

The table below provides a brief description of the purpose and duration of each study visit for the delayed OBVAT group.

Visit	Purpose	Main Procedures	Duration

IRB#: 2210026053 APPROVED: 20-Oct-2023 EXP: 19-Oct-2024

**Visit 1***	**Screening visit**	**Screening consent, interview, medical record review, vision assessment, informed consent, randomization, assignment to SCC+OBVAT or SCC alone.**	**30 minutes or until all questions about the study are answered**
**Visit 2-3***	**Initial Assessment**	**Optometric clinical exam, vision tasks with cardiovascular, questionnaires**	**Either one 90 minute visit with a break OR**<br>**One 60 minute visit (visit 2) and one 30 minute visit (visit 3)**
**Visit 4**	**Initial imaging assessment**	**MRI**	**60 minutes**
**Visit 5**	**Outcome visit 1**	**Optometric clinical exam, vision tasks wtih cardiovascular, questionnaires**	**60-75 minutes with break**
**Visit 6**	**Outcome visit 1 Imaging assessment**	**MRI session**	**60 minutes**
**Visits 7-22**	**Therapy visits**	**60 minute visit with vision therapy procedures**	**60 minutes**
**Visit 23**	**Outcome visit 2**	**Optometric clinical exam, vision tasks with cardiovascular, MRI, questionnaires**	**60-75 minutes with break**
**Visit 24**	**Outcome visit 2 Imaging assessment**	**MRI**	**60 minutes**
**Visit 25**	**Outcome visit 3 (about one year later)**	**Optometric clinical exam, vision tasks with cardiovascular, questionnaires**	**60-75 minutes with break**
**Visit 25**	**Outcome visit 3 (about one year later)**	**MRI session**	**60 minutes**

***=The screening visit and initial assessment may be done on the same day if timing and schedules allow**

What will be done with my data during this study?

During the study, we will collect data from you. By agreeing to participate in the study, you agree to give this data to New Jersey Institute of Technology for research purposes.

Will I receive any results from the tests done as part of this study?

Results that could be important for your clinical care will be shared with you. We will not share other results with you.

What are the risks of this study?

Taking part in a research study involves inconveniences and risks. The main risks of taking part in this study are discussed below.

Risks of Eye exam:

There is a risk that the eye exam may temporarily provoke or worsen your concussion symptoms, but it will not make your brain injury worse. The exam may be stopped any time if you start to feel uncomfortable.

Risks associated with OBVAT and home reinforcement sessions:

IRB#: 2210026053 APPROVED: 20-Oct-2023 EXP: 19-Oct-2024

There are no known medical risks associated with OBVAT. However, you may experience mild discomfort or exacerbation of concussion symptoms which should resolve within a few hours.

Risks of MRI:

There are no known medical risks associated with MRI. However, you may feel uncomfortable inside the magnet if you do not like to be inside small places or have difficulty lying still. You will be provided a squeeze ball alarm as well as a voice intercom to tell us if you would like to stop the MRI scan. MRI machines produce loud banging noises, which cause some people to become stressed or upset. You will be required to wear ear plugs to reduce the noise of the MRI scan.

The MRI magnet is always on and attracts certain metal objects. Any metal objects on or inside of your body may heat up, move, and/or not function properly within the scanning room. Metal objects in the room can fly through the air toward the magnet and hit those nearby. There are many safety measures in place to reduce these risks. The staff will screen all persons and materials entering the scanning room for metal. When the study begins, the door to the room will be closed to minimize the risk of someone accidentally bringing a metal object into the scanner room.

Risks of Vision Tasks and Eye Tracking and Cardiovascular:

There are no known risks to the vision tasks you will complete in this study. However, you may experience mild visual discomfort due to eye strain or fatigue, or may experience worsening of concussion symptoms which should resolve within a few hours. While it is unlikely that the goggles worn during these tasks may cause discomfort, if you feel any discomfort or irritation from the goggles, you may take a break or stop the activity. You will have electrodes attached that are like stickers, a cuff placed on your arm and finger to measure blood pressure, cardon dioxide sensor, and doppler probe on head/neck.

Risks of Questionnaires

There is a risk that the questionnaires may make you feel uncomfortable. You will not have to answer any questions that you do not want to.

Risks associate with breach of confidentiality:

As with any study involving collection of data, there is the possibility of breach of confidentiality of data. Every precaution will be taken to secure participants' personal information to ensure confidentiality. At the time of participation, each participant will be assigned a study identification number. This number will be used on data collection forms and in the database instead of names and other private information. A separate list will be maintained that will links each participant's name to the study identification number for future reference and communication.

Are there any benefits to taking part in this study?

You might benefit in the form of a decrease in concussion or visual symptoms as a result of participating in OBVAT. However, we cannot guarantee or promise that you will receive any direct benefit by participating in this study. The knowledge gained from this research may help doctors determine the effect of OBVAT on recovery after concussion and may help improve care for concussion patients.

Do you need to give your consent in order to participate?

If you decide to participate in this study, you must sign this form. A copy will be given to you to keep as a record.

What are your responsibilities?

Please consider the study time commitments and responsibilities as a research subject when making your decision about participating in this study. You will need to follow the study doctor's instructions, keep all study appointments and complete the OBVAT therapy as directed.

What happens if you decide not to take part in this study?

Participation in this study is voluntary. You do not have to take part in order to receive care at Somerset Pediatric Group.

If you decide not to take part or if you change your mind later there will be no penalties or loss of any benefits to which you are otherwise entitled.

Can you stop your participation in the study early?

You can stop being in the study at any time. You do not have to give a reason.

IRB#: 2210026053 APPROVED: 20-Oct-2023 EXP: 19-Oct-2024

Can the study doctor take you out of the study early?

The study doctor may take you off of the study if:

- Your condition worsens.
- The study is stopped.
- You cannot meet all the requirements of the study.
- New information suggests taking part in the study may not be in your best interests.

What choices do you have other than this study?

There are options for you other than this study including:

- Receiving vision and concussion care outside this study.
- Not participating in this study.
- You may discuss other options available to you with your doctor.

What about privacy, authorization for use of Personal Health Information (PHI) and confidentiality?

As part of this research, health information about you will be collected. This will include information from medical records, assessments, questionnaires and tests. Information related to your medical care will go in your medical record. This could include physical exams or imaging studies (MRI scans). Medical records are available to New Jersey Institute of Technology staff. Staff will view your records only when required as part of their job. Staff are required to keep your information private. Information that could identify you will not be shared with anyone - unless you provide your written consent, or it is required or allowed by law. MRI results will appear in your medical record. We will do our best to keep your personal information private and confidential. However, we cannot guarantee absolute confidentiality. Your personal information may be disclosed if required by law.

The results of this study may be shown at meetings and published in journals to inform other doctors and health professionals. We will keep your identity private in any publication or presentation.

Several people and organizations may review or receive your identifiable information. They will need this information to conduct the research, to assure the quality of the data, or to analyze the data or samples. These groups include:

- Members of the research team and other authorized staff at the New Jersey Institue of Technology.
- Researchers at the The Children's Hospital of Philadelphia since they are working on this study with us.
- Researchers at the Pennsylvania College of Optometry.
- The Imaging Coordinating Center at Rutgers University.
- The Eye Institute of Salus University since they are working on this study with us.
- People from agencies and organizations that perform independent accreditation and/or oversight of research; such as the Department of Health and Human Services, Office for Human Research Protections.
- The National Institutes of Health who is sponsoring this research.
- If you receive payment using a bankcard or check payable to the person participating in the study, the bank will have access to identifiable information. The bank will not have access to any medical information.

By law, the New Jersey Institute of Technology is required to protect your health information. The research staff will only allow access to your health information to the groups listed above. By signing this document, you are authorizing New Jersey Institute of Technology to use and/or release your health information for this research. Some of the organizations listed above may not be required to protect your information under Federal privacy laws. If permitted by law, they may be allowed to share it with others without your permission.

There is no set time for destroying the information that will be collected for this study. Your permission to use and share the information and data from this study will continue until the research study ends and will not expire. Researchers continue to analyze data for many years and it is not possible to know when they will be completely done.

Certificate of Confidentiality (CoC)

A Certificate of Confidentiality (CoC) covers this research. A CoC helps protect your identifiable information and biological samples.

A CoC protects your private information from all legal proceedings. Unless you consent, information from this research study that identifies you will not be shared outside this research.

- No one can be forced to share your identifiable information for a lawsuit.
- Your information can't be used as evidence even if there is a court subpoena.

If you consent, your data could be shared for:

IRB#: 2210026053 APPROVED: 20-Oct-2023 EXP: 19-Oct-2024

- Other scientific research

The CoC does not prevent some disclosures.

- The researchers can't refuse requests for information from those funding this research. The NIH may need information to assess this project.
- The US Food and Drug Administration (FDA) may need information.
- You can still share information about yourself. You can also freely discuss your involvement in this research.
- The researchers must disclose things required by law. This includes suspected child abuse and neglect, harm to self or others, or communicable diseases.

Can you change your mind about the use of personal information?

You may change your mind and withdraw your permission to use and disclose your health information at any time. To take back your permission, it is preferred that you inform the investigator in writing.

Dr. Tara Alvarez
New Jersey Institute of Technology
323 Martin Luther King Boulevard
Fenster Hall Room 611
Newark, NJ 07102

In the letter, state that you changed your mind and do not want any more of your health information collected. The personal information that has been collected already will be used if necessary for the research. No new information will be collected. If you withdraw your permission to use your personal health information, you will be withdrawn from the study.

Financial Information

Your health insurance or you will not be billed for any procedures (vision exam, objective eye movement recordings, imaging or therapy visits) that are part of this research project.

Will there be any additional costs?

There will be no additional costs to you by taking part in this study.

The NIH is providing financial support and material for this study and all research devices/assessments.

Will you be paid for taking part in this study?

- You will be paid up to $400 for your time and effort in the form of a bankcard or a check. You will be given $100 at the end of the initial assessment, $100 at the completion of outcome visit 1, and $200 at the completion of outcome visit 2. You must complete all OBVAT sessions and assessments to receive full compensation for participation in the study.
- You will be paid up to $200 for your time for an approximate one year follow-up assessment which is the optometric clinical exam, vision test with cardiovascular measures and imaging session.

Who is funding this research study?

The National Institutes of Health is providing funding for this study.

What if you have questions about the study?

If you have questions about this study or how your data is going to be used, call the study primary investigator, Tara Alvarez at 973-596-5272. You may also talk to your own doctor if you have questions or concerns.

The Institutional Review Board (IRB) at New Jersey Institute of Technology has reviewed and approved this study. The IRB looks at research studies like these and makes sure research subjects' rights and welfare are protected. If you have questions about your rights or if you have a complaint, you can reach the IRB Office at irb.njit.edu.

A description of this clinical trial will be available on http://www.ClinicalTrials.gov, as required by U.S. Law. This Web site will not include information that can identify you. At most, the Web site will include a summary of the results. You can search this Web site at any time.

IRB#: 2210026053 APPROVED: 20-Oct-2023 EXP: 19-Oct-2024

Sharing Data with the National Institutes of Health (NIH)

Why will my data be shared with the National Institutes of Health (NIH)?

The NIH is funding this study. The NIH's goal is to maximize the benefits that come from the research.

The NIH repository stores phenotypic data from many studies. The NIH then shares that information with researchers. We will send the information about you and the other participants to a repository at the NIH. The information will be de-identified (no names or other direct information about you will be included). The NIH will not be able to re-identify you or any other individual.

The NIH intends to share the collected information with other researchers for future research. The researchers who receive data must promise to keep the data confidential and to use it only for the purpose approved by NIH. They must also promise to not try to re-identify anyone.

Risks Associated with Sharing Data with the NIH

There are risks associated with sharing your data with the NIH but they are very unlikely to occur. There is only a very small chance that someone could find out that the data came from you. If that happened, it's possible that someone could deny you a job or health insurance. Or you could experience stress, anxiety or embarrassment.

Benefits Associated with Sharing Data with the NIH

Sharing your information for future research will not directly benefit you. It is hoped that it will lead to knowledge that could help others in the future.

Controlled or Unrestricted Access

The data about you will either be made available by the NIH through controlled access or unrestricted. Controlled access means the data are made available for other research only after investigators have obtained approval from NIH to use the requested data for a particular project. Data for unrestricted access are publicly available to anyone (e.g., The 1000 Genomes Project).

1) **Consent to Share Data with the NIH**

Please indicate whether you will allow us to share your information with the NIH by check the box next to one of the following choices:

* must provide value

- ◯ YES I do consent to sharing my de-identified information with the NIH
- ◯ NO I do not consent to sharing my de-identified information with the NIH

Consent for Use of Data for Future Research

As part of the study, we will collect information from your medical records and the study procedures that you complete. We may wish to use your data in future studies about concussion. Your data will be given a unique code and will not include information that can identify you. Information that can identify you may be kept permanently in a secure database at New Jersey Institute of Technology. Only the study doctors and those working with them on this study will be able to see information that can identify you.

Your identified data will be retained only if you agree to the future use of your data or to be contacted for future studies. If you leave the study, you can ask to have the data collected about you removed. You can also ask us to remove information that identifies you from the data.

Please indicate whether you will allow the data to be used for future research by checking the box next to one of the following choices:

2) **Consent for Use of Data for Future Research**

* must provide value

- ◯ NO my data may be used for this study only.
- ◯ YES my data may be used for other future research studies. If the data are shared outside of New Jersey Institute of Technology, only a limited data set (no identifiers except the dates of your visits to Somerset Pediatric Group) will be included.

IRB#: 2210026053 APPROVED: 20-Oct-2023 EXP: 19-Oct-2024

Consent to Take Part in this Research Study and Authorization to Use and Disclose Health Information for the Research

The research study and consent form have been explained to you by:

3) **Name of Person Obtaining Consent**

* must provide value

4) **Signature of Person Obtaining Consent**

* must provide value

5) **Date of Person Obtaining Consent** 11-23-2022 M-D-Y

* must provide value

By signing this form, you are indicating that you have had your questions answered, you agree to take part in this research study and you are legally authorized to consent to your child's participation. You are also authorizing the use of your/your child's health information as discussed above. If you don't agree to the collection, use and sharing of health information, you cannot participate in this study.
NOTE: *A foster parent is not legally authorized to consent for a foster child's participation.*

6) **Subject First Name**

* must provide value

7) **Subject Middle Name**

* must provide value

8) **Subject Last Name**

* must provide value

9) **Subject Age**

* must provide value

10) **Subject Consent (18 years or older)**

○ I consent to participate in the study

○ I do not consent to participate in the study

By checking the consent box, you are indicating that you have had your questions answered and you agree to take part in this research study.

11) **Signature of Subject**

12) **Date of Consent** 11-23-2022 M-D-Y

IRB#: 2210026053 APPROVED: 20-Oct-2023 EXP: 19-Oct-2024

13) Parent/Legal Guardian Consent

◯ I consent to have my child participate in the study

◯ I do not consent to have my child participate in the study

By checking the consent box, you are indicating that you have had your questions answered, you agree to take part in this research study, and you are legally authorized to consent to your child's participation. You are also agreeing to let CHOP use and share your child's health information as explained above. If you don't agree to the collection, use, and sharing of your child's health information, your child cannot participate in this study. NOTE: a foster parent is not legally authorized to consent for a foster child's participation.

14) Name of Parent/Legal Guardian

15) Signature of Parent/Legal Guardian

16) Date of Consent M-D-Y

Child Assent to Take Part in this Research Study

For children capable of providing assent:

I have explained this study and the procedures involved to the subject in terms he/she could understand and that he/she freely assented to take part in this study.

17) Name of Person Obtaining Assent

18) Signature of Person Obtaining Assent

19) Date of Person Obtaining Assent M-D-Y

20) Assent (Subjects under 18 years old)

◯ I assent to participate in the study

◯ I do not assent to participate in the study

By checking the assent box, you are indicating that you have had all of your questions answered and you agree to take part in this research study.

21) Signature of Subject

22) Date of Assent 11-23-2022 M-D-Y

23) Would you like a signed copy of the consent form emailed to you?

◯ Yes

◯ No

If not, a standard copy of the consent form will be sent to you with study contact information for your reference.

Next Page >>

IRB#: 2210026053 APPROVED: 20-Oct-2023 EXP: 19-Oct-2024
